# Automatic Updates of Transition Potential Matrices in Dempster-Shafer Networks Based on Evidence Inputs

**DOI:** 10.3390/s20133727

**Published:** 2020-07-03

**Authors:** Joel Dunham, Eric Johnson, Eric Feron, Brian German

**Affiliations:** 1 Aerospace Engineering, Georgia Institute of Technology, Atlanta, GA 30332, USA; eric.feron@aerospace.gatech.edu (E.F.); brian.german@aerospace.gatech.edu (B.G.); 2 Aerospace Engineering, Pennsylvania State University, University Park, PA 16801, USA; eric.johnson@psu.edu

**Keywords:** Dempster-Shafer, valuation network, joint conditional matrix, optimization, least squares, transition potential, reasoning under uncertainty

## Abstract

Sensor fusion is a topic central to aerospace engineering and is particularly applicable to unmanned aerial systems (UAS). Evidential Reasoning, also known as Dempster-Shafer theory, is used heavily in sensor fusion for detection classification. High computing requirements typically limit use on small UAS platforms. Valuation networks, the general name given to evidential reasoning networks by Shenoy, provides a means to reduce computing requirements through knowledge structure. However, these networks use conditional probabilities or transition potential matrices to describe the relationships between nodes, which typically require expert information to define and update. This paper proposes and tests a novel method to learn these transition potential matrices based on evidence injected at nodes. Novel refinements to the method are also introduced, demonstrating improvements in capturing the relationships between the node belief distributions. Finally, novel rules are introduced and tested for evidence weighting at nodes during simultaneous evidence injections, correctly balancing the injected evidenced used to learn the transition potential matrices. Together, these methods enable updating a Dempster-Shafer network with significantly less user input, thereby making these networks more useful for scenarios in which sufficient information concerning relationships between nodes is not known *a priori*.

## 1. Introduction

Sensor fusion is a topic central to aerospace engineering; this topic is particularly applicable to unmanned aerial systems (UAS) and autonomous systems that rely on sensors to either provide remote information to an operator or provide information that forms the basis of automated decision-making. Evidential Reasoning, also known as Dempster-Shafer theory [1,2], is used heavily in sensor fusion for classification of detections [3,4]. However, Dempster-Shafer (DS) implementations generally have high computing requirements [4], leading to limitations in use on small UAS platforms. Valuation networks [5,6], the general name given to evidential reasoning networks by Shenoy, provides a means to reduce computing requirements through knowledge structure. However, these networks typically either use the Transferable Belief Model (TBM) to reduce relationships between nodes in the network to conditional probabilities used in a Bayesian network [3,6], or they use joint conditional probability matrices, originally termed transition potential matrices by Shenoy [7]. TBM is based on non-probabilistic belief function theory [8], in contrast to the probability-based belief function theory that underpins DS Theory [8]. Transition potential matrices have significant data requirements, which typically require expert information to define and update.

This paper proposes and tests a novel method to learn these transition potential matrices based on evidence injected at nodes. Novel refinements to the method are also introduced, demonstrating improvements in capturing the relationships between the node belief distributions. Finally, novel rules are introduced and tested for evidence weighting at nodes during simultaneous evidence injections, correctly balancing the injected evidence used to learn the transition potential matrices. Together, these methods enable updating a Dempster-Shafer network with significantly less user input, thereby making these networks more useful for scenarios in which sufficient information concerning relationships between nodes is unknown *a priori*.

This paper is structured as follows:

- Section 2 provides a brief overview of Dempster-Shafer theory
- Section 2.3 provides a brief overview of evidence propagation through a network
- Section 3.1 discusses the requirements of this network to combine evidence at each node
- Section 3.2 develops novel rules to update the transition potential matrices based on evidence inputs to nodes
- Section 4 develops the novel use of episodic learning for Dempster-Shafer networks, improving the transition update results
- Section 5 develops novel rules for evidential weighting to combine evidence in the network
- Section 6 shows tests and results for the novel update rules
- Section 7 discusses the conclusions, limitations, and future work for the novel transition potential matrix update methods

## 2. Background: Dempster-Shafer Theory

Dempster-Shafer (DS) theory was originally devised by Arthur Dempster [1] and Glenn Shafer [2]. The following papers provide a mathematical background to the theory [4,9]. “Smart Projectile State Estimation Using Evidence Theory” provides a practical understanding of evidence theory using sensor fusion and state estimation as the backdrop [10]. Other practical explanations of DS theory are available [11]. For the purpose of this work, focusing on extensions to DS theory as applied to networks, this background includes a simple, practical example to set the stage for understanding what the DS network offers. This example will be related to Bayesian reasoning for readers familiar with that framework. Readers who are already familiar with DS theory and its complications can jump to Section 2.3.

### 2.1. Dempster-Shafer Information Fusion Example

**Figure 1:**
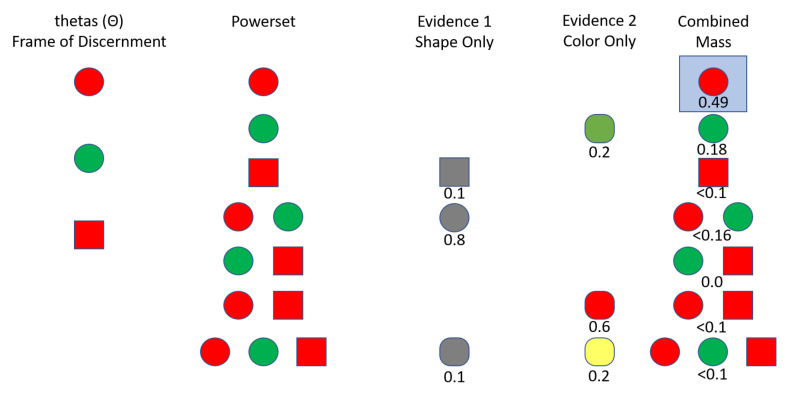
Visual representation of the simple Dempster-Shafer example. Θ represents the three options that could be observed by the sensors. The powerset column represents all combinations, which Dempster-Shafer analysis considers. Recall that sets with multiple options signifies the belief that the observed object could be any one of the objects in the set. The two sensors that provide observations—evidence—can detect all objects, but can only detect certain properties of each object. Those detections are shown, along with the belief masses assigned to each element of the powerset—the Basic Probability Assignment (BPA). The combined mass column shows the powerset again, along with the results of the analysis which correspond to the greater details in Table 1. The highlighted element of the powerset (the red ball) is the θ which is believed to correspond to the true object, based on the Dempster-Shafer analysis.

Using the defined nomenclature (see Nomenclature), this section focuses on a simple example which will make the DS theory and application clearer. Consider a situation in which an object is one of the following options as shown in Figure 1.

- Red ball
- Green ball
- Red cube

Together, these options comprise the Frame of Discernment, Θ, as the object under question can only be one of these. The powerset of Θ is then shown in the “Powerset” column of Table 1. Assume two sensors provide evidence concerning the object. Evidence one is provided by a black and white camera that can only distinguish shape but with error. Evidence two is provided by a sensor that only distinguishes color with error. Suppose the object in question is a red ball. The evidence provided by sensor one may look similar to the belief masses in the “Evidence 1” of Table 1. The “Evidence 1” column is then a Basic Probability Assignment (BPA) assigning the belief masses to each element in the powerset. All elements with non-zero mass are Focal Points. This evidence can be interpreted as the sensor is 10% sure the object is a cube, 80% sure the object is a ball, and 10% unsure of what the object is.

The “Evidence 2” column represents a potential set of evidence from a sensor that only distinguishes color. In this case, sensor two is 20% sure the object is green, 60% sure the object is red, and 20% unsure of the color. This sensor is less precise at distinguishing colors than sensor one is at distinguishing shapes. Dempster’s Rule [1] is used to combine the evidence, and the result is shown in the “Combined” column of Table 1.

Notice that the unknown element—the complete set—is significantly reduced in the combined dataset from each of the two evidence sets. Since the evidence sets were not highly conflicting, unknowns and ambiguities (sets that include more than one θ but not the complete set) were reduced. Also, note that the correct classification, the red ball, has the highest combined mass of any of the elements of the powerset. Looking at the “Bel” and “Pl” columns of Table 1—the belief and plausibility functions, respectively—one can see that the belief functions are the sums of all masses that could apply to that element, and the plausibility functions are one minus the sum of all masses that could not apply to that element. Thus, the belief function for a red ball equals the combined mass for a red ball while the belief function for a red ball and a green ball is equal to the sum of the belief masses for a red ball, a green ball, and a (red ball, green ball). Likewise, the plausibility function for a red ball is one minus the sum of the belief masses for a green ball, a red cube, and a (green ball, red cube). Looking at the difference between the belief function and plausibility function columns leads to a few conclusions:

- This example concludes that there is a precise understanding of the belief associated with the object being either a red ball or a red cube because the belief and plausibility function values—the “Bel” and “Pl” values for the (red ball, red cube) row in Table 1, which represent the lower and upper bounds on the belief—are nearly the same value.
- There is a similar precise understanding of the belief associated with the object being either a red ball or a green ball, but the belief in that case is significantly higher than the belief that the object is either a red ball or a red cube. This result is expected given the sensor setup for the example.
- The largest range of belief values is associated with the correct object classification—the red ball. This classification is also the strongest belief and highest plausibility of any of the θ elements.

Finally, we take a look at this same problem from a Bayesian perspective [12]. Before doing this, we must note one nuance concerning the DS approach. The DS approach had an original unstated hypothesis concerning the object: that the type of object was unknown (all belief mass is assigned to the complete set). When Dempster’s rule is used to combine a BPA of all mass assigned to the complete set with another BPA, the result is the same as the second BPA, thus allowing that step to be removed from the simple example. For a comparable Bayesian example, we start with all mass equally divided among the Θ elements, using similar terminology as the DS example for ease of comparison. The first point to note is that the closest representation of unknown in Bayesian is equal probability distribution across all options. However, this distribution is indistinguishable between equal probabilities of all options being correct versus no knowledge of which option is correct. The Bayesian observations are then cube versus ball for the first evidence and red versus green for the second evidence. The ambiguities in the DS evidence translate into likelihoods (correct and incorrect) for the Bayesian test, and we assume the observation is correct—a red ball. The result of these computations is shown in Table 2. From this analysis, we can make a few observations:

- The correct answer—a red ball—has the highest posterior probability (the final column in Table 2), meaning that the object in question is most likely a red ball.
- The evidence was highly supportive of the correct answer to the extent in the DS example that the belief and plausibility functions of the Θ elements do not overlap. The Bayesian result does not communicate this information at all, meaning that in the Bayesian analysis, the decision-maker cannot be as confident in the decision that the object is a red ball.

In summary, this simple example resulted in the same outcome with Bayesian and DS analyses. However, the additional information contained in the DS analysis presents the reliability of the results to the decision-maker. For a decision based on the highest probability, either analysis works. However, suppose for example that the decision-maker wants to ensure that the object in question is not a red cube or a green ball. In the Bayesian analysis, there is no way to know the upper limit on the probability for the object either being a red cube or a green ball. From Table 1, in the DS analysis, the upper limit of the probability that the object is a green ball is 0.367. The upper limit on the probability that the object is a red cube is 0.163. The lower limit that the object is a red ball is 0.490. Thus, for a decision-maker whose responsibility is ensuring that the object is not a red cube or a green ball, the DS analysis provides the information necessary to make that decision. The Bayesian analysis does not provide the required information. This is one reason why DS analysis is used heavily in risk analysis and sensor fusion, typically for classification of sensed objects.

### 2.2. Dempster-Shafer Combination Rules

With this basis in understanding of the application of DS analysis, the next step is to look at the combination rules. The original DS combination rule is linear, which preserves the axioms required for probability combinations [1], thus allowing this method to reduce to Bayesian reasoning under certain conditions. However, this rule suffers from a couple of issues, the principle one being the assumption that all evidence contributors have equal weight, reliability, and full knowledge of the frame of discernment. In our simple example in Section 2.1, each sensor still had full knowledge of all elements of the powerset (each sensor can detect every possible combination), but returned ambiguities when it could not distinguish between specific θs. This application adheres to the assumptions of Dempster’s Rule. In contrast, suppose that sensor one could not detect red cubes. In that case, sensor one would always assign zero mass to any subset of the powerset that contains red cubes. This situation violates the assumptions of Dempster’s Rule and leads to non-intuitive results due to the classic vote-no-by-one issue in which a single no-vote by an evidence contributor results in the option being assigned a belief of zero when the evidence is combined. For highly conflicting data, this results in non-intuitive results such as in Table 3 [4].

Because the assumptions of Dempster’s Rule are often not applicable to real-life scenarios (in most situations, all evidence observations do not have full knowledge of all elements of the powerset and are not equally reliable), multiple authors since the 1980s have devised ways around these assumptions including different combination rules as well as different input functions that add unknown mass to account for the lack of knowledge of elements of the powerset. Since this paper is not an overview of these combination rules, the focus will be placed on three combination rules that have useful properties for the analysis being developed. Zhang’s combination method [13], Murphy’s combination method [14], and the Evidential Reasoning rule [15] are three such methods that enable a custom weighting to be associated with each new evidence. This property will be useful later in the development of the network in Section 5.

### 2.3. Evidence Propagation

**Figure 2:**
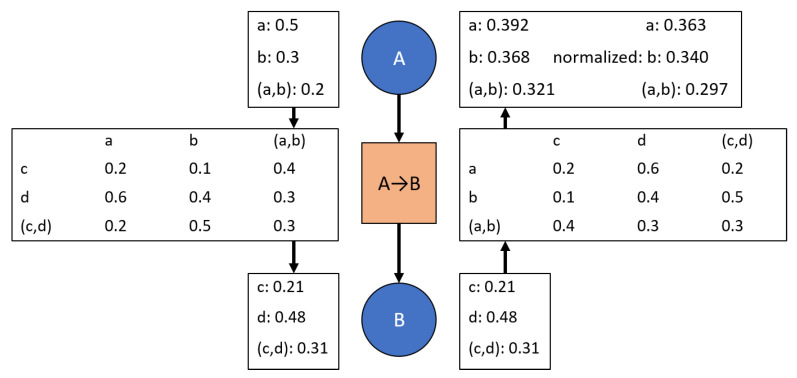
Propagating the Dempster-Shafer evidence masses through the transition between nodes. For simplicity, only two options are available at each node. The direction of propagation is represented by the arrows between evidence masses. The arrows between the nodes represent the definition of the network. As can be seen, evidence propagation from node A to node B results in normalized masses. Conversely, evidence propagation from node B to node A results in non-normalized masses. Moreover, the original masses are not recovered if masses are propagated from node A to node B to node A through the same transition.

Evidence propagation through a hypertree [7] was first introduced in the 1980s. Similar to Bayesian propagation, the principle difference is that evidence propagation is less concerned with the transition values being conditional probabilities. This is best shown through Figure 2, which shows a parent node with its transition to a child node. Note that it is easy to convert transition values into conditional probabilities [6,7]. Since DS reasoning condenses down to Bayesian reasoning under certain assumptions [16], evidence propagation is very similar to Bayesian propagation. In fact, the example given in the original work by Shafer and Shenoy is a Bayesian example that overwrites nodes with new evidence and treats conditional probabilities at each transition as a known fact used for evidence propagation [7]. In order to maintain consistency in the network with known conditional probabilities, it is necessary to overwrite node information with marginal probabilities inferred through the conditional transitions based on the most recent evidence update. The effect of this can also be seen through Figure 2 since propagating even an identical input to the previous child marginals does not result in the parent marginals being recovered. This mechanism assumes that the most recent evidence is the best choice and, therefore, limits the capabilities for the network to incorporate uncertain evidence at each node.

This original work was extended through the 1990s and early 2000s under various names for the field of study. Valuation networks is the general name given to these networks by Shenoy [5], which are not required to be directed (i.e., each connection between nodes has a direction associated with it) or acyclic (i.e., given any starting node, there are no paths in the network following the directions between nodes that return to the starting node), and they encode the relationships between characterizations of the uncertainty for local sets of knowledge. The determination of whether sets of knowledge can be broken into separate nodes is based on conditional independence [17], for example, if sets of knowledge are conditionally independent from each other, then they can be broken into separate nodes with the relationship encoded on the link between the nodes. Uncertainty propagation was extended by Smets [6,18] based on the Transferable Belief Model (TBM). While the Transferable Belief Model is a powerful tool for capturing the relationships between knowledge sets, it is based on the non-probabilistic belief function theory [8], in contrast to the probability-based belief function theory that underpins DS Theory [8]. Using the formal theory of TBM, Smets showed the relationships between the joint probabilities used in Shenoy’s original work [7] and conditional probabilities used in Bayesian networks [6]. Specifically, Smets showed that conditional probabilities for each of the θs in node *A* that are conditionally dependent on node *B* could fully capture the effects of the probability distribution of node *B* on the θs in node *A* [6]. This relationship allowed the joint probabilities from Shafer and Shenoy [7] to be reduced to the minimum representation of conditional probabilities, which results in reduced computer memory usage and could leverage conditional probability calculations already developed for Bayesian networks. This work applies to valuation networks in general—no directed acyclic assumptions required—and has since been extended [19].

**Figure 3:**
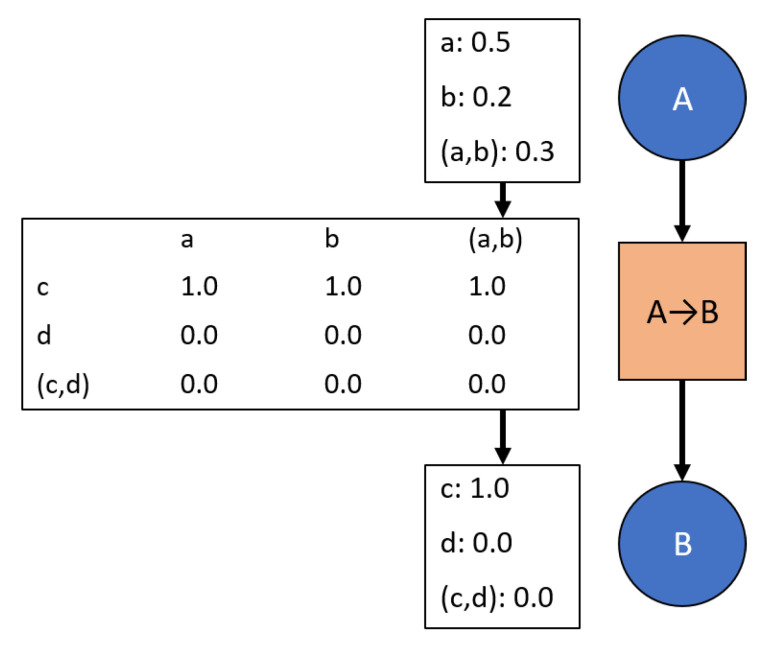
An example case in which the conditional probabilities are a minimum, and a smaller, representation of the joint probabilities. The variable *d* in node *B* is not influenced at all by the variables *a* and *b* in node *A* and is, thus, independent from the variables in node *A*. The conditional probabilities would reflect that relationship by eliminating the middle row of zeros, resulting in fewer values being stored to represent the relationship.

More recently, primarily in the 2000s, work has returned to the joint probabilities originally used by Shafer and Shenoy [7] for encoding the relationships between nodes in a valuation network. Evidential networks—valuation networks which use evidential reasoning to combine observations at nodes and joint mass tables (the general case of joint probabilities as seen in Figure 2)—have been extended and applied to various scenarios including threat assessment [3,8,20]. Further, discounting has been introduced to reduce the weight of inferred evidence versus directly observed evidence [3]. These extensions and applications are consistent with the original work [7] while offering improvements in computation speed, reliability weighting of evidence, and analysis of what evidence can be propagated between nodes. However, these extensions are still based on the same assumptions about joint masses—the joint masses are set and updated occasionally by someone who has knowledge of the relationships between nodes. This concept of unchanging joint masses or joint probabilities is an assumption that allows the joint probabilities to be represented as conditional probabilities. This assumption clearly underlies the use of vector projections and spans to show that the conditional probabilities are a minimal representation (i.e., minimum information required) of the joint probabilities [6]. Consider the case in Figure 3 in which variable *d* is not influenced by the variables in node *A*, thus allowing the conditional probability representation to be smaller than the joint probability representation.

While this assumption is useful if the joint probabilities are not changing often, the application of the evidential network in this paper is to unmanned systems and sensor systems, and an underlying assumption of this analysis is that the relationships between nodes are constantly changing as operational data is received. Thus, the move from joint probabilities to conditional probabilities actually causes an issue.

This model of joint probabilities also demonstrates the significant data requirements mentioned in Section 1. Consider the joint probabilities in a two node network linking a node with two θs to a second node with two θs, which is the minimum size for a Dempster-Shafer network. In this case, there are three values in each node’s powerset, resulting in 3∗3=9 values that the expert has to define and update in the joint probabilities. For higher numbers of θs, this data requirement grows quickly. A five θ node linked to a five θ node results in a joint probabilities matrix with 31∗31=961 values to be updated.

Published papers that are based on evidential reasoning networks often assume a directed acyclic network, such as the work by Pollard and Pannetier [3], which can model the knowledge required for the applications described in this paper. Further, directed acyclic networks with multi-path loops can always be reconstructed to remove the loops. Since directed acyclic networks are sufficient for the decision analysis in this paper, directed acyclic networks are assumed for the rest of the developments in this paper.

Other methods of handling valuation network representations have been developed [21,22]. While these representations have less in common with the evidential reasoning networks, limitations are introduced to prevent information from being incorrectly inferred [21]. For example, if the network is comprised of two nodes such that the parent node has two options: “writer” and “not writer”, and the child node has two options: “journalist” and “not journalist”, evidence suggesting the entity under question is not a journalist cannot be used to infer evidence against the entity being a writer.

### 2.4. Parameter Update Methods

Many well-researched methods currently exist for automatically updating parameters. In particular, meta-heuristic algorithms such as Simulated Annealing, Generic Algorithms, and the Cross-Entropy Method have been used for parameter optimization [23]. Further, with untethered communications and cloud-based services often available, cyber-physical systems can be used to significantly increase computing power and data storage available for sensor parameter optimization, enabling optimization methods such as meta-heuristic algorithms to be performed on big data [24]. In contrast, UAS often operate with limited communications beyond the onboard system [25,26]. Further, especially for small UAS, onboard computing power is often limited and primarily used for mission control systems [25]. Thus, solution methods analyzed for this paper, as discussed in Section 3.2, were focused on optimization methods that can potentially run on an onboard system with limited computation power and limited data storage with predictable execution times. Several optimization methods were analyzed, as discussed in Appendix C, and were suggested for future research due to less predictable execution times or less reliable implementations.

## 3. Transition Potentials Updates Based on Evidence

**Figure 4:**
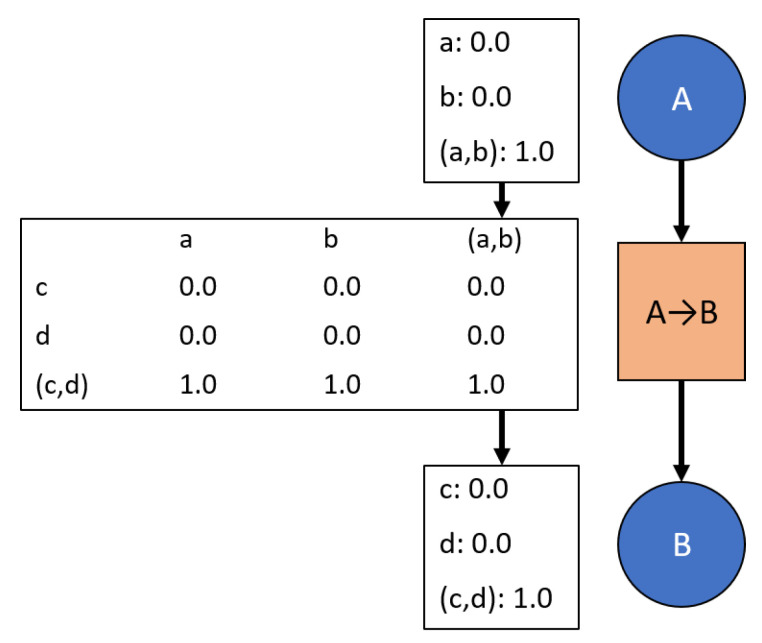
An example network that starts with no data. This analysis is beyond the scope of Bayesian logic since Bayesian requires priors. Further, overwriting information is risky in this context since a full overwrite of information suggests sufficient data behind each update. In other words, the first overwrite would be similar to starting with Bayesian logic after the first update, but insufficient information for that event to occur has already been assumed.

Current rules for updating the network have difficulties with highly limited *a priori* data because the transition potentials are only updated based on user inputs, thus requiring sufficient knowledge of the relationships between nodes before the network is used. New rules have been developed to facilitate intuitive evidence propagation when transitions between nodes start with limited or unknown information. Unknown transition information is represented as transitions mapping all marginal masses to the complete set as shown in Figure 4. This state is referred to as “vacuous” [6]—providing no information on the joint probabilities between the nodes.

### 3.1. Evidence Combination at Nodes

One of the principle strengths of DS theory is combining evidence in a single frame of discernment. Developing a hypertree or network enables structuring the data such that each local frame of discernment is computationally feasible. For example, as in Figure 2, each node has 2 options, which means each powerset comprises 3 options (see Nomenclature for powerset definition). Without the structure, there are 4 options available, which means a powerset of 10. As the number of nodes and hypotheses $\left(\theta s\right)$ per node grow, an evidential reasoning network quickly becomes the only computationally feasible option for limited computing power. In addition, information about causality between hypotheses is obfuscated. Within a network, however, new evidence provided at each node often still includes uncertainty and should be combined with previous evidence at that node [3,27]. This is especially true for evidence inferred through a transition from another node, which can be considered to be less reliable than directly observed evidence.

**Figure 5:**
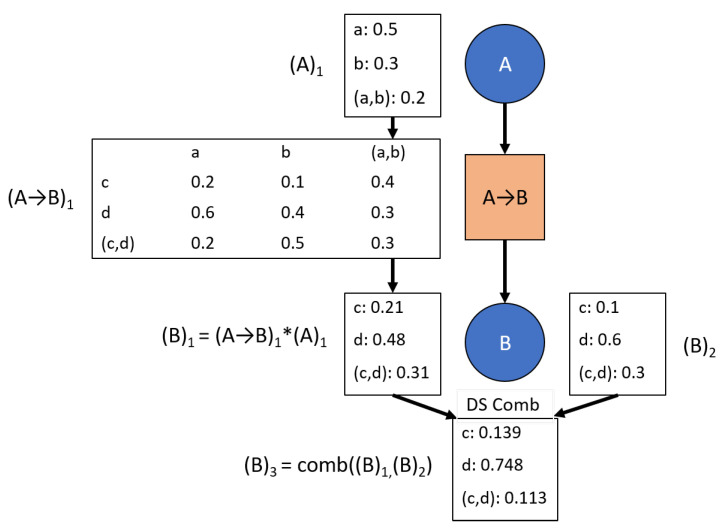
An example network update that uses Dempster-Shafer updates to combine evidence at each node. The combined value from node A ${\left(A\right)}_{1}$ is propagated as evidence through the transition to node B ${\left(B\right)}_{1}$, where it is combined with directly injected evidence ${\left(B\right)}_{2}$, resulting in ${\left(B\right)}_{3}$.

The first rule defined for this network is that all evidence updates at each node uses an evidential combination rule, of which there are multiple available [1,13,14,15,28,29] as shown in Figure 5. This rule aligns with previous work [3,27]. The inherent drawback to using combination methods to update each node is immediately realized in that the network values are no longer consistent with each other once evidence has been propagated through a transition and combined at a node—that is, the node’s marginal probabilities no longer equal the previous node’s marginal probabilities multiplied by the transitional values, which can be clearly seen in Figure 5. This result suggests the possibility of learning the transitional values through updating the transitions to be consistent with the new node values. This update is discussed in Section 3.2.

### 3.2. Transition Updates

As stated in Section 3.1, subsequent to combining evidence at a node, the network is no longer locally consistent across transitions to the neighboring nodes. This state leads to the hypothesis that the transition can be updated to return to a consistent state. The transition is governed by the following three sets of Equations (1)–(3), assuming the transition matrix is *T*, the parent marginal column vector is $M_{p}$ with *p* values, and the child marginal column vector is $M_{c}$ with *c* values.

(1) $$M_{c}=T*M_{p}$$

(2) $$\sum_{i=1}^{c}{\left(col_{j}\left(T\right)\right)}_{i}=1\;\forall \;j=1,\ldots ,p$$

(3) $$T_{i,j}\geq 0\;\forall \;i=1,\ldots ,c\;\mathrm{and}\;j=1,\ldots ,p.$$

Due to Equations (3) and (2), all values are automatically limited to the range $\left[0,1\right]$, which is necessary for transition potentials, per definition [7]. These equations do not fully constrain the transition potentials, thus requiring an optimization routine to choose a feasible solution, which minimizes a cost function. The equality constraints (Equations (1) and (2)) can be rewritten into a vector equation of the form Ax=b, where *A* is a matrix and *b* and *x* are vectors. This equation can be optimized to solve for *x*. There are two important points in this optimization design:

- In most cases, there will be one redundant equation. The redundant equation is not known *a priori* because it is dependent on the parent and child marginals. Therefore, all equations are included in the optimization, which does not degrade the solution.
- The inequality constraint, Equation (3), is not included in the vector equations. Depending on the optimization routine and cost function chosen, this constraint may or may not be included.

The primary goal is to find a feasible solution which updates the network in a stable manner and is quickly computable. With that aim in mind, the cost function was chosen to be Equation (4).

(4) $$min\left(\sum_{i,j=0,0}^{i,j=m,n}{\left(T_{i,j}^{*}-T_{i,j}\right)}^{2}\right).$$

This cost function minimizes the change from the previous transition matrix to satisfy the stable update goal. While other cost functions can be chosen, this cost function enables least squares optimization, which does not require iteration and satisfies the goal of fast computations. Note that other solution methods may be able to meet the stated goals, as discussed in Appendix C. It is clear, however, that the cost function is incompatible with the design vector. This issue is because the design vector, *x*, is based on Equations (1) through (3), but Equation (4) is the difference between the previous transition matrix and the new transition matrix. Thus, the design vector is modified to account for this difference, with the resulting offsets calculated from the previous transition matrix and added into the *b* vector. Importantly, a least squares solution without constraint modifications does not guarantee all design variables are greater than zero, which is necessary to satisfy Equation (3). In practice, the cost function pushes the design variables towards positive semi-definite values and usually provides feasible solutions since the previous values are positive semi-definite. This shortcut was deemed necessary to improve the solution speed. However, when the least squares solution returns an infeasible solution, another method has to be chosen.

The least squares solution always meets the constraints given by Equations (1) and (2) since those are defined for the solution method, and it was previously shown that at least one solution always exists that meets all constraints. The task is then to adjust the solution minimally to meet the constraints defined in Equation (3). This adjustment can be done through a series of mathematical operations defined below that (i) maintain adherence to the constraints in Equations (1) and (2), (ii) find a result, which meets the constraints in Equation (3), and (iii) attempts to minimize deviations from the solution found via the least squares method. The goal is to modify all values that are negative to be non-negative. Due to the constraints given in Equation (2), this automatically guarantees that any values greater than the value of one will also be reduced to less than or equal to the value of one. The procedure is as follows:

(1) Order the columns to adjust values. Do this by summing all values that are less than zero or greater than one in the column and sort from greatest to least. This order is useful because the maximum value this sum can attain is 1.0. This is because any value above 1.0 must have an equivalent set of values below zero to compensate. However, values below zero can be balanced by values in the range (0.0,1.0]. Since this is true, when the sum is equal to 1.0, the scenario is the most highly constrained in which all negative values must be used to balance the value that is greater than 1.0. Note that any columns that already meet the constraint will sum to 0.0.
(2) Step through each column from step (1) above in order.
(3) For each column, order all values in the column that are less than 0.0 from maximum absolute value to minimum absolute value. While this order is not required, it provides a consistent solution method which makes debugging easier.
(4) For each value in order from step (3) above, redistribute excess mass per Algorithm 1.

**Algorithm 1:** Mass redistribution algorithm. This algorithm redistributes mass, which does not adhere to the final constraint in Equation (3) in a way that seeks to minimize the difference from the minimum solution found through the least squares approach.
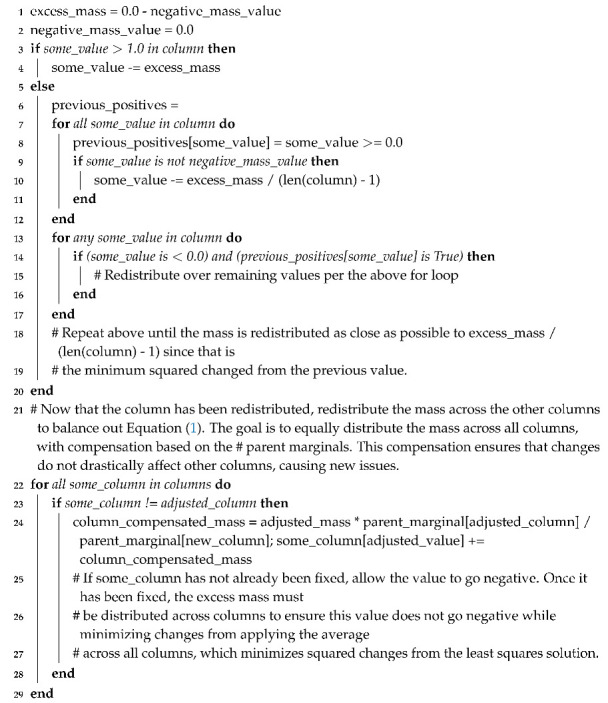


**Figure 6:**
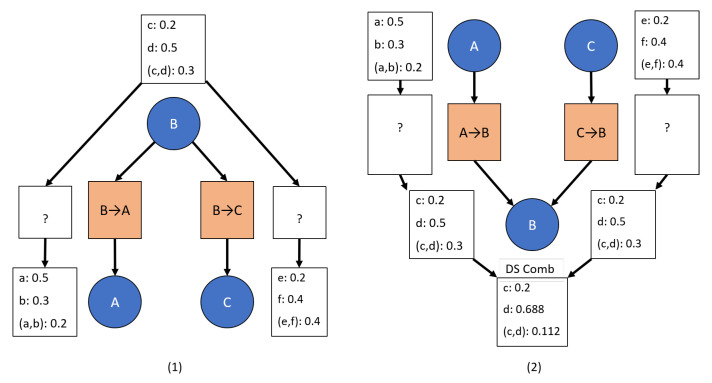
Case (**1**) shows a single parent node with transitions to two child nodes. Each unknown transition can be calculated using the update method described previously via either least squares minimization or linear programming minimization. Moreover, case (**1**) reduces to the simplest case of one parent and one child node if node C and the associated transitions are removed. In contrast, case (**2**) cannot solely be solved via the described least squares or linear programming methods. The child marginal values are a result of a Dempster-Shafer combination algorithm, which must be part of the method for updating the unknown transition potentials. This case is handled in Section 3.3.

As shown in Figure 6, this method can handle cases in which the child node of the transition being updated only has a single parent transition—case (1) in Figure 6. For multiple parent transitions—case (2) in Figure 6—this update method is too simplistic since each parent transition would return the child marginals, with the combination of those marginals resulting in a different solution.

This effect means that the transition potentials that link multiple parent transitions to the same child node must be solved together. The naïve approach is to simultaneously solve for all transition potentials using Equations (2) and (3). Equation (1) would be modified to Equation (5).

(5) $$M_{c}=comb\left(T_{1}*M_{p1},T_{2}*M_{p2},\ldots ,T_{n}*M_{pw}\right).$$

In Equation (5), comb is the DS combination algorithm chosen from the list of options discussed previously, and *w* is the number of parent transitions that must be simultaneously solved. The primary issue with this method is that DS combination methods can have highly non-linear effects depending on the evidence sets fed into the combination method. Further, because all transition potentials are being solved simultaneously, the optimization routine becomes significantly more complex to run, causing a large, non-deterministic increase in optimization time. To combat these issues with this solution method, two simplifying assumptions are made:

- The simultaneous optimizer is used only to solve for the DS combination of marginals that results in the child marginals. This problem is significantly smaller than optimizing the full transition matrices with the DS combination algorithm included. Then, the solution methods for individual branches (the previously-described least squares and linear programming minimization methods) is used to find the correct transition potentials for each parent transition based on the specific marginals for that branch.
- With the assumptions that the DS network is initialized with completely unknown information and all transitions are learned as information is added to the network, then there is no *a priori* information concerning the transition potentials. Thus, any solutions that meet the previously defined constraints of transition potentials are reasonable. Given this, the following simplifying assumption is made when updating the transition potential matrix: all marginals combined via a DS algorithm to produce the desired child marginal are the same. Subsequently, this assumption can be relaxed for certain combination algorithms.

Given these two assumptions, an optimizer could be used on the reduced problem to find the marginals which combine to create the desired child marginal. An appropriate optimizer would be a trust-region interior points method or something similar. However, this optimization still suffers from non-deterministic run times and difficulty sectioning the optimization routine for real-time operating systems unless a specific optimization routine were written for this. Instead, a more reliable and faster method was developed for certain combination algorithms as detailed in Section 3.3.

### 3.3. Multi-Parent Transition Updates

Several DS combination algorithms reduce to the same algorithm when combining identical evidence sets. In particular, the original DS rule [1], Murphy’s rule [14], Zhang’s rule [13], and Evidential Combination Reasoning (ECR) [15] are the same for identical evidence sets and equal weights. This result is because (i) Murphy’s rule and Zhang’s rule reform the evidence masses per their rules, then combine the reformed evidence masses via Dempster’s rule $\left(n-1\right)$ times, where *n* is the number of evidence sets; and (ii) ECR was specifically designed to reduce to Dempster’s Rule when using equal weights [15]. Because Dempster’s rule does not result in a normalized combined evidence set regardless of whether the input evidence sets are all normalized, Yager’s rule [29], which is similar to Dempster’s rule but assigns the unallocated mass to the universal set, does not fit the previous pattern, and the result developed in this section does not apply to Yager’s rule [29]. While this result may seem fairly constrained since it only applies to four rules, it serves well for most decision networks since those four rules can cover the various cases to which decision networks are typically applied. The averages in Murphy’s rule [14] apply when low beliefs of an event occurring are important. Conversely, Zhang’s rule [13] applies when outliers need to be eliminated. The original DS rule [1] and ECR [15] apply when only the combined evidence is known at each update; the history of evidential inputs is not retained. Further, by combining Zhang’s rule [13] with sufficient evidence history, the winning decision will be emphasized, which is one of the primary propositions of the Rayleigh methods [28]. Finally, the chief property of Yager’s rule [29]—avoiding issues with Dempster’s rule [4]—is also a property of Murphy’s rule [14], Zhang’s rule [13], and ECR [15].

To develop the algorithm, first note that in Dempster’s rule, input evidence masses can only apply to output masses that are a subset of the input mass. For example, $\left(a,b,c\right)$ applies to all elements of the powerset, but $\left(a,b\right)$ can only apply to *a*, *b*, and $\left(a,b\right)$. Thus, for identical input evidence sets, the only contributor to the universal set (in the example—$\left(a,b,c\right)$) is the universal set, to the *n* power, where *n* is the number of times the identical evidence sets are combined. With no other dependencies, the input mass for the universal set is immediately solvable from Equation (6).

(6) $${\left(universal\;set\right)}_{out}={\left(universal\;set\right)}_{in}^{n}.$$

Continuing on with this trend, an example for two identical evidence set inputs and 3 options per input is shown in Table 4.

When converted to equation form, the equations for each of the combined evidence masses is a polynomial of order *n*, where *n* is equal to the number of identical evidence sets entered into the combination algorithm. Further, each combined evidence mass is only dependent on inputs of the same evidence mass and on evidence mass inputs closer to the universal set. For example, in Table 4, the resulting mass $a_{out}$ is only dependent on $a_{in}$, ${\left(a,b\right)}_{in}$, ${\left(a,c\right)}_{in}$, and ${\left(a,b,c\right)}_{in}$. This set of polynomial equations can be solved individually if done in the correct order, which results in an easy algorithm to run as well as one that can be paused mid-update for real-time operating systems.

Expanding this example further, the general case is shown in Equations (7) through (8), where *o* means out, *i* means in, *n* is the number of input evidence sets, universalset−p is the subsets of number of elements of the universal set minus *p* elements, and $g_{\left(\ldots \right)}$ are the multipliers for each polynomial term and follow the pattern defined in Algorithm 2.

(7) $${\left(universal\;set\right)}_{o}={\left(universal\;set\right)}_{i}^{n},$$

(8) $$\begin{array}{c} \forall m_{o}\in {\left(universal\;set-p\right)}_{o}\;m_{o}=\\ m_{i}^{p}+g_{p-1}{\left(\sum \forall q_{i}\in \left(universal\;set-r\right),r=\left[0,p-1\right]\;\&\;q_{i}\cap m_{i}=m_{i}\right)}_{i}^{1}m_{i}^{p-1}\\ +\cdots +g_{1}{\left(\sum \forall q_{i}\in \left(universal\;set-r\right),r=\left[0,p-1\right]\;\&\;q_{i}\cap m_{i}=m_{i}\right)}_{i}^{p-1}m_{i}^{1}\\ +\left(\sum q_{i}\in \left(universal\;set-r\right),r=\left[0,p-1\right]\;\&\;q_{i_{1}}\cap q_{i_{2}}\cap \cdots \cap q_{i_{n}}=m_{i}\right). \end{array}$$

**Algorithm 2:** Multiplier calculation algorithm. The polynomial multipliers are based on the equations detailed above. This algorithm provides a simple method for calculating these multipliers in code.

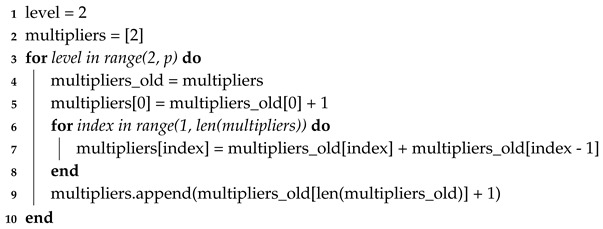



Recalling that all focal points $m_{i}$ must be positive semi-definite, the equation above presents a bound on the capabilities of this solution. Specifically, the zero-eth power term in Equation (8) must be less than or equal to the child marginal, $m_{o}$. If this is not the case, then the equation returns a negative solution. Assuming that the child marginal is a combination of identical parent marginals—as in the case of Murphy’s Rule or Zhang’s Rule—of at least the number of parents, then a valid solution will always be found via this method. In practice, this limitation is not an issue. This limitation simply means that the child node must have enough evidences combined to be at least the number of parent nodes. In effect, the solution method cannot guarantee a valid solution until the node is sufficiently initialized. In many cases before the initialization is complete, a valid solution is available even though it is not guaranteed. Within the initialization, the case in which all parents but one have the total mass in the complete set is trivial, since the remaining parent can be set equal to the child. Other situations only arise when using Dempster’s Rule or ECR. Currently, a solution is not available to calculate the parent marginals without using a high dimensionality optimization, which is a slow process. Instead, the focus is placed on restricting the inputs to ensure that the child marginals have an available solution for the parents. This restriction is accomplished by analyzing the polynomial solution discussed in Equations (7) through (8). The restrictions in Equations (9) and (10) allow Dempster’s Rule and ECR to retain the ability for the reverse calculation for multiple parents, assuming that the previous assumption of the existence at least as many inputs as parents holds true. Validation of this restriction is shown in Appendix A.

(9) $$\forall m_{a_{in}}\;except\;universal\;set,m_{in}\leq m_{b_{in}}s.t.m_{b_{in}}\subset m_{a_{in}},$$

(10) $$m_{{\left(universal\;set\right)}_{in}}\leq \sum m_{{\left(a,\ldots \right)}_{p-1}}\;where\;p=len\left(universal\;set\right)\;and\;a\subset \Theta .$$

By combining the equations and algorithms developed in this section with those in Section 3.2, updates to the nodes in the DS network can be used to learn the transition potentials between all nodes in the network. While these updates can be performed when a single node is observed and other nodes are updated based on inference, more reliable updates are performed when more than one node is observed simultaneously. However, these updates present a conundrum since each node update will propagate throughout the entire network, thus providing the effect of several updates simultaneously. The solution to this issue lies in proper weighting of the updates, which is discussed in Section 5.

## 4. Episodic Learning

As shown in Section 6, the learning methods detailed in Section 3 enable the DS network to understand the relationship between nodes based on evidential inputs at each node. This method is sufficient for some applications: when the windowed mass distribution at each node represents the entire mass distribution of interest for understanding the network relationships. Recall that the window can be defined as long as appropriate for the application. With reasonable weighting (see Section 5), the window can encompass the entirety of the evidence history.

**Figure 7:**
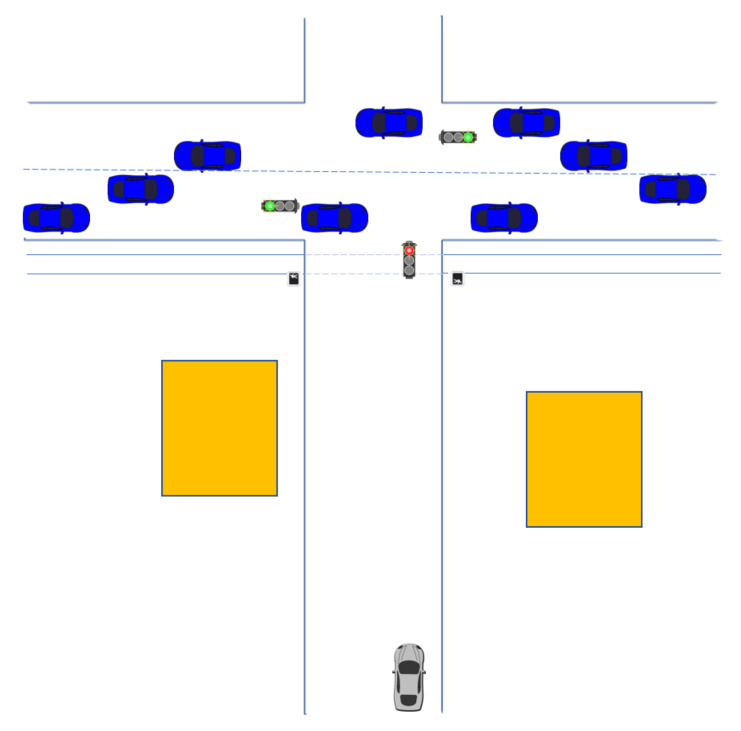
The layout for the traffic signal scenario, which is representative of timed four-way lights without left turn signals. The grey car is approaching a red light intersection and estimating how long until the light turns green to determine whether to slow the car. Visibility is limited due to buildings and other obstructions. The cross-walk signal may be visible before the intersection. The cross-traffic light is not visible to the grey vehicle and must be estimated. Cross traffic density and speed is variable in the simulation and is estimated by the grey vehicle.

However, this view is limited. In many cases, the entire mass distribution is not visible in the current window, and including the entirety of the evidence history does not capture the nuances of the relationships. For example, consider a traffic light scenario as shown in Figure 7 in which the network is evaluating the relationship between the time until the light turns green and the state of the cross-traffic light. The relationship would be roughly expected as shown in Table 5.

The issue with learning these relationships is that the entire distribution is not present at a given time. When the cross-traffic light is green, estimates of time until the same-side light changes from red to green are likely only long and medium. Likewise, when the cross-traffic light changes to red, estimates of time until the same-side light changes from red to green are likely only short and medium. Using the previously developed learning methodology in Section 3, the natural response would be to retain all evidence via Murphy’s rule using a weighting scheme. However, this method will be biased by the amount of time spent in each situation. For example, assuming the light is being evaluated from a long distance away approaching the intersection, one can safely assume that the evidence gathered usually reports the cross-traffic light as green and the time until the same-side light changes from red to green as long. In this case, the learned relationship will be biased towards a long time until green regardless of the current state of the cross-traffic light, because the majority of evidence is during a period in which it takes a long time for the light to change to green (i.e., other relationships are masked by the amount of evidence showing a long time to green, even though much of this evidence is just repeating known information). In order to combat this issue, an episodic learning method was developed, which required two additional mathematical rules.

### 4.1. Change-Weighted Least Squares

Recall from Section 4 that the goal is to capture in the transition the nuances of the relationship between the mass distributions of the parent and child nodes. Note that while this discussion is applied to a single parent and child, it can be generalized to multiple parent or child nodes using the techniques developed in Section 3. The question then becomes how to mathematically capture the concept of only updating the weights in the transition that apply to the current episode. Suppose the baseline distributions for the parent and child nodes are known. With no loss of generality, we will take those baseline distributions as the unknown distribution: p=0∀p∈M∧p≠universalset and p=1⇔p=universalset, where *M* is the marginal mass vector of the parent or child. Then, modify Equation (4) to include weighting relative to the change from the baseline distribution, as shown in Equation (11), where *c* is an arbitrary control term for determining the effect of the weighting. Note that Equation (1) is modified with the weights defined in Equation (11) to balance the equations.

(11) $$min\left(\sum_{i,j=0,0}^{i,j=m,n}{\left(\left(T_{i,j}^{*}-T_{i,j}\right)*\left(1+\frac{c}{{\left(M_{p_{i}}^{*}-M_{p_{i}}\right)}^{2}*{\left(M_{c_{j}}^{*}-M_{c_{j}}\right)}^{2}}\right)\right)}^{2}\right).$$

Observe three points concerning Equation (11):

- If the control constant *c* is set to 0, then the additional weight disappears, and the equations reduce to the previous set.
- The weight is inversely proportional to the squares of both the differences in applicable parent marginals and the applicable child marginals. If the child or parent marginal changes minimally, then the design vector is weighted such that the applicable transition value should be modified minimally from its current value.
- If either the parent or child marginal change is zero, then the inverse weighting is undefined—divide by zero scenario. Thus, this weighting must be protected by a maximum weight. In practice, any weight on the order of 1000 or above does not have much effect since there is a limit to the flexibility of the solution given the rest of the constraints.

### 4.2. Zero Marginal Value

Recall from Equations (1) to (3) that a zero marginal value in the child marginal vector means that all non-zero values in the parent marginal vector must be multiplied by a transition value of zero for the equations to balance. Assuming marginal probabilities are reset to a baseline between episodes in order to capture the effects of each episode, it is reasonable to assume that marginal values will often be zero even if those values were observed in prior episodes. In practice, this can cause prior episodic information to be lost. To avoid this loss of information, any marginal value, which was observed in a prior episode is prevented from returning to zero and is instead reset to a small value in the range of [0,1], such as 0.01. Due to the weighting described in Section 4.1, such a small value will result in a high weight, preventing the associated transition values from changing significantly.

### 4.3. Episodic Learning Implementation

Given the additional mathematical rules described above, episodic learning is implemented as shown in Algorithm 3. Determining episodes can be accomplished either through expert information or automatically by determining when marginals are statistically different than previous episodes.
**Algorithm 3:** Episodic learning algorithm for a Dempster-Shafer network. This algorithm captures episodes in order to only update the portion of the transition potential matrix to which the episode applies.
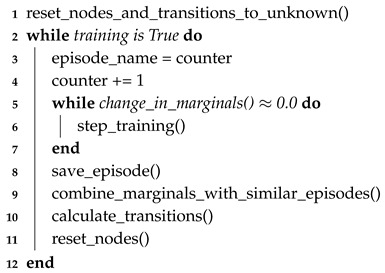


Evaluation of this method is detailed in Section 6.6, which compares the results of the learning methods with a simplified scenario for evaluation against expected results.

## 5. Evidence Weight

**Figure 8:**
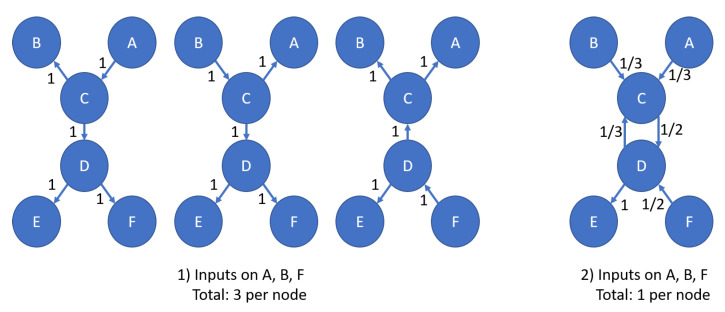
The results of weighting inputs for a Dempster-Shafer network. Given an update on nodes A, B, and F, a no-weight update results in 3 times the amount of experience applied at each node, as shown in part (**1**) of the figure. With weighting, only the applicable experience is applied at each node, as shown in part (**2**) of the figure. The direction of the arrows shows the transition of the update experience between nodes in the network. The network is using the standard representation, where moving upwards on the diagram between nodes represents inference.

Traditional DS evidence combination assumes equal weights and reliabilities between all evidence sets and contributors [1]. Likewise, most modifications to the DS combination rule make the same assumption. An exception is the Evidential Reasoning rule which handles both reliability and weight of evidence sets [15]. While a single DS node handles the assumption of equal weights well, the network requires the ability to weight evidence. Recall that from Section 2.3, “evidence” propagated between nodes in the network is really the result of combined data at nodes; thus, individual evidence weighting within nodes has already occurred. Weighting the combined evidence propagated through the network is the current focus and requirement. To understand this requirement, observe the update to the network in Figure 8. Assuming that all inputs propagate through the network, each input equally affects all nodes, although the data is inserted as observed at a single node and inferred at others. Further, all observations may be a result of a single update (for example, the results of a one-hour test flight). Each input would result in an update at all nodes, introducing the equivalent of *y* hours of test flight data, where *y* is the number of observations for the network from the one-hour test flight. While more node observations for the one-hour test flight likely results in more accurate information, the data arguably should not have the same effect as multiple hour test flights with observations for each of those hours. Note that this discrepancy was previously handled by a discounting scheme to handle the reduced reliability of inferred evidence versus directly observed evidence [3]. This scheme is similar to Shafer discounting [2]. While useful, no rules were developed to define the discounting factor [3]. Further, discounting has additional effects on evidence combination [4], which must be handled. This section develops a new rule that explicitly defines the weighting factor based on the network input evidence, and the weighting factor can be calculated explicitly from the network for each update. Further, weighting methods (discussed in Appendix B) are explicitly developed for each combination rule used, avoiding known issues with Shafer discounting [15].

To resolve this discrepancy between the weight of evidence and the time of observation relative to pre-existing data, a weighting scheme is proposed, as demonstrated in Figure 8, part (2). At observed nodes, only the observation is entered with full weight. No data calculated through transitions is entered at observed nodes. For all other nodes, the weight of data calculated through transitions is one divided by the number of branches with data entering that node. The resulting weight for a single update is equal to one for all nodes.

Use of the weighting scheme has two implications. First, either only the Evidential Reasoning combination method can be used, or other methods must be modified to handle weighting. Second, given that weighting is necessary, further weighting methods can be used to improve update stability as discussed subsequently. Modifications for weights are easy to add to Murphy’s Rule, Zhang’s combination method, and the Rayleigh combination methods. Details on these modifications can be found in Appendix B.

Table 6 shows why additional weighting schemes are necessary since a single event, regardless of the length of operation time it represents, can significantly impact the per-hour risk if weights are not adjusted for the evidence sets. Since the combination scheme impacts the results as well, the Rayleigh and Murphy results are still significantly different regardless of weighting method. Consequently, risk analysis results thrash between extremes as differing events are entered into the network. Instead, a weighting scheme based on completed flight hours is defined. For each network node, the accumulated hours of experience represented by the combined evidence is stored. New evidence sets are relatively weighted as $\frac{operation\;hours}{total\;operation\;hours}$. This concept is similar to how new values are added into an average: by multiplying the average by the total experience it represents and adding the new values before dividing by the new total experience. In both cases, the weighting scheme prevents new evidence from over-influencing previous experience.

Two further available weighting modifications were tested and found to be less effective, although they are reasonable options.

- Recency: new evidence can be weighted higher than the relative weight to the total experience for risk analysis in which more recent information is considered more reliable.
- Inference: an additional weight reduction can be applied across all transition inferences assuming that inferred evidence is less reliable than directly observed evidence. This method has been used previously [3], although the weights are explicitly now defined for each Evidential Reasoning combination method.

Finally, this method does assume equal importance between nodes. If nodes are unequal in their importance or reliability, then additional weight reductions can be applied to account for those imbalances, similar to the inference modification detailed above.

## 6. Results

A DS network was developed to test the algorithms developed in Section 3. Testing covered the following areas:

- Single node updates with and without learning transitions, and with and without weighting. These tests include modifying the transition potentials, learning from a completely unknown starting point, and multi-level learning (i.e., with unobserved nodes between nodes with observations). See Section 6.3.
- Multiple parent updates with weighting and learning transitions. See Section 6.4.
- A complex network to better understand the speed and effects of evidence propagation in a more realistic DS network. See Section 6.5.

### 6.1. Testing Methods

For each test, evidence sets were randomly generated. The same evidence sets and order of input were used for all combination algorithms at each test to provide consistency. Each test consisted of 30 updates of 3 simultaneous evidence sets per update. The nodes to which the evidence sets were injected were randomly generated as well. The number 30 was chosen to allow randomly generated sets to provide acceptable analysis metrics within a reasonable evaluation time. Furthermore, 30 tests were conducted per test case. For each evidence set, the complete set (unknown information) mass was set to 0.0, which ensured that the learning capabilities of the networks were properly evaluated against the metrics discussed in Section 6.2. For cases without learning, approximately 80% of transition potential values were randomly set, since, without learning, the majority of transitions need to be set for the network to make sense. For learning cases, all values were defaulted to all belief mass assigned to the complete set (i.e., completely unknown starting points). The primary limitation of these tests is that the evidence sets are randomly generated. DS theory is designed to combine evidence sets for a given situation to arrive at a conclusion. As such, some consistency between the evidence sets is assumed. In fact, complete conflict, as discussed in Section 2, produces incalculable results for Dempster’s Rule and the ECR rule without weighting. Due to round-off error and pseudo-random selection of evidence sets, it was assumed that complete conflict would not occur. However, since combination algorithms handle conflict differently, results are generally only valid when analyzed in comparison between tests using the same combination algorithm.

### 6.2. Metrics

The DS network extensions developed in this work were evaluated for the following goals: propagation time for implementation in real-time on an embedded system, learning for the ability to start from a state with completely unknown information, and explainability for the decisions to be accepted by organizations that use risk analysis.

- Propagation time per update per node: comparison between the baseline implementation and specific novel additions.
- Failures: how many of the tests fail, and the explanation for each failure.
- Consistency: the L2 norm of the error between the parent marginals multiplied by the transition potentials and the child marginals. Note that for a multi-parent node, the results of the parent marginals multiplied by the transition potentials are then combined via the appropriate DS combination algorithm.
- Unknown fraction: the fraction of node marginals and transition potentials that remain in the complete set (unknown information) at the end of the update set, given that the network started with all masses in the complete (unknown) set.
- Weight per node: the total weight/experience attributed to each node at the end of every update.

The propagation time metric provides insight into the ability to implement in real-time on an embedded system. Likewise, the unknown fraction metric provides insight into the learning capability of the network and the ability to start from an unknown state. Less intuitive are the consistency and weight per node metrics, which provide insight into the explainability of the network. Weight per node maps the “experience” per node after the updates. For example, if each update represents one flight test hour, and 30 updates are made, each node should show approximately 30 h of experience. Significant increases from that suggest that information is used more than once, which calls into question the validity of any conclusions.

The final metric—consistency—evaluates the mathematical explainability of the network at the end of all updates. Given a simple network where a parent node “A” connects to a child node “B”, an inconsistent situation is one in which, after evidence propagation through the network, the marginals of “A” multiplied by the transition potentials do not equal the marginals of “B”. From an explainability perspective, if only the final network state is viewed by the organization that needs to accept the decision, it is unclear from where the decision came and whether the network operated correctly. In short, the result is unexplainable. Since the original DS network implementation overwrites data at each node after propagation, an “overwrite” algorithm is included as the baseline for comparison.

### 6.3. Network Updates with Single Parent Only

**Figure 9:**
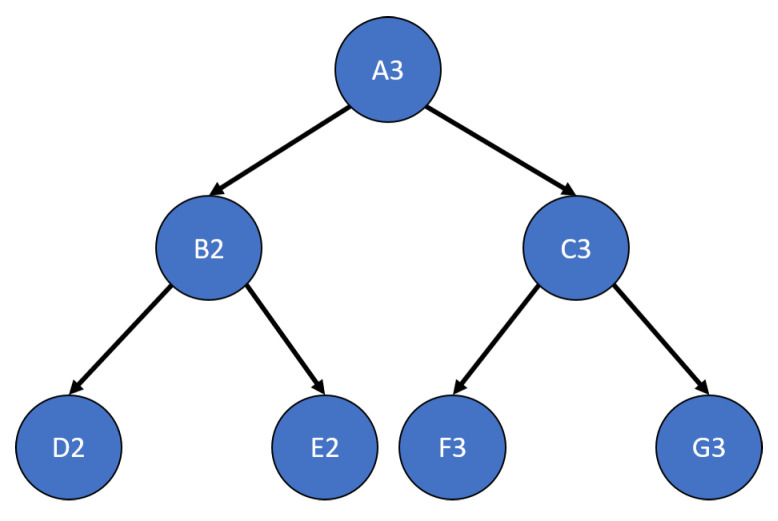
Test network used to analyze the performance of the novel Dempster-Shafer network algorithms. Only includes single parents for each node. The number after the node name shows the number of θs for the node. Nodes with two and three θ were used since these are the more common cases for nodes in a DS network.

This test analyzes the performance of the developed algorithms when applied to networks that only have single parents per child. The test network is shown in Figure 9. Each DS combination algorithm was analyzed using this test network including an “overwrite” algorithm that does not combine evidence but rather uses the newest evidence set to provide a baseline. This test case analyzed the following options: learning transitions, single versus multi-node updates, and weighting inputs.

**Figure 10:**
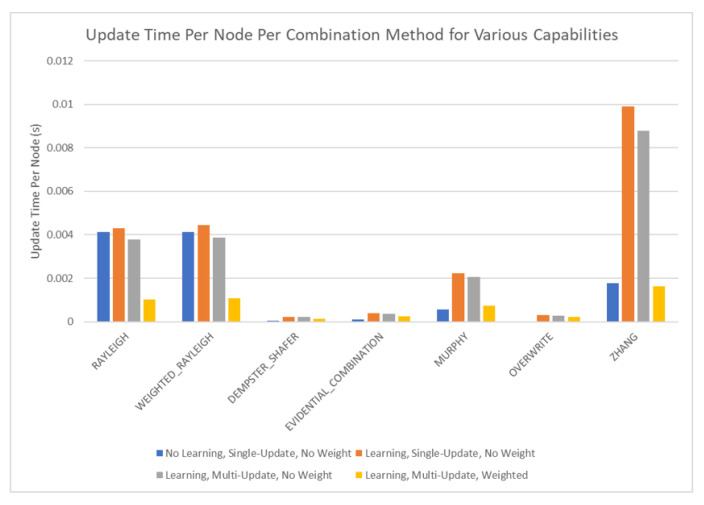
Test results for run time with single parent network. Within each combination algorithm group, the no-learning method is faster that un-weighted learning methods, showing the increased burden of the learning calculations. Both Rayleigh and Weighted Rayleigh methods show the same order of magnitude for no-learning time and un-weighted learning time, suggesting that the combination algorithm is the driving factor for the update time. Dempster-Shafer, Evidential Combination Reasoning (ECR), and Overwrite have faster update times due to not retaining explicit history. Note further that un-weighted single and un-weighted multi-update methods do not significantly change the update times. Finally, the weighted methods are typically at least as fast as the no-learning method.

**Figure 11:**
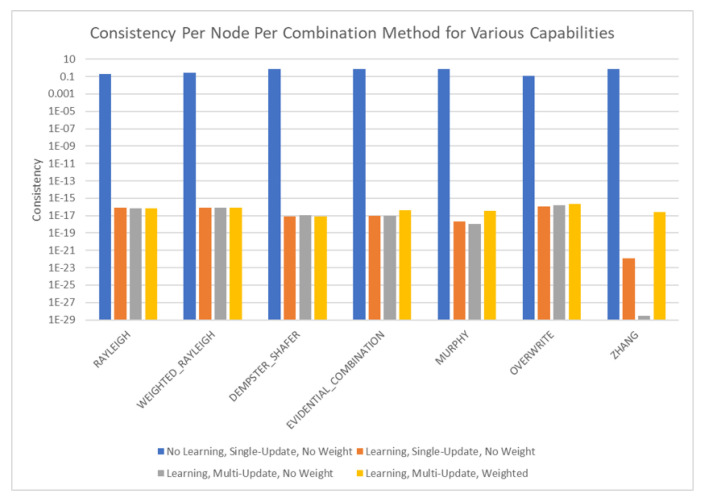
Test results for consistency with single parent networks. The y-axis uses a logarithmic scale. All per-node consistency is low without learning, with the best case being the overwrite case. Since transition potential matrices are not reversible (propagating up through a transition does not guarantee perfect consistency down the transition), the overwrite case has a non-zero consistency test result. However, in all learning cases, the consistency checks return an effectively zero result, equating to perfect consistency with round-off error.

**Figure 12:**
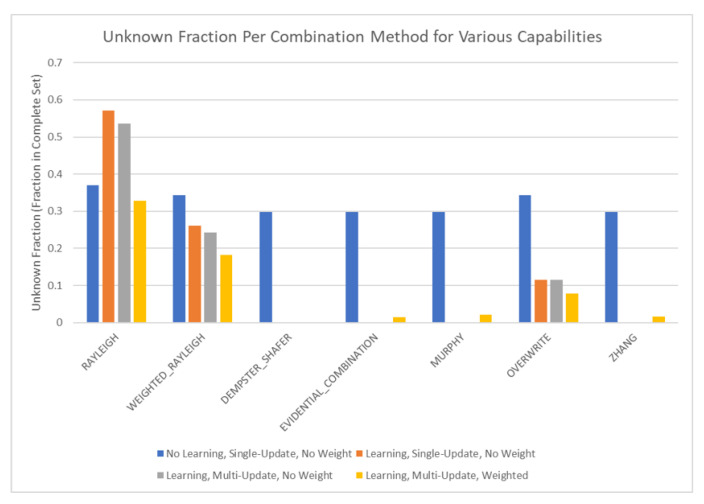
Test results for learning with a single parent network. Results vary depending on the combination algorithm used. Except for Rayleigh, Weighted Rayleigh, and overwrite methods, the learning method resulted in significant reduction of unknown information over the baseline case of no-learning with preset transition potentials. Deviations in the combination algorithms can be explained through handling of conflict. High conflict in the Rayleigh and Weighted Rayleigh algorithms means lower assignment of mass to the new focal elements, resulting in higher mass retained in the unknown/complete set. This is a result of the randomized evidence set testing methodology and does not impact the choice of algorithm. Finally, since the overwrite method does not retain previous evidence, propagated evidence through unknown transitions will tend to have higher impact, retaining unknown information.

**Figure 13:**
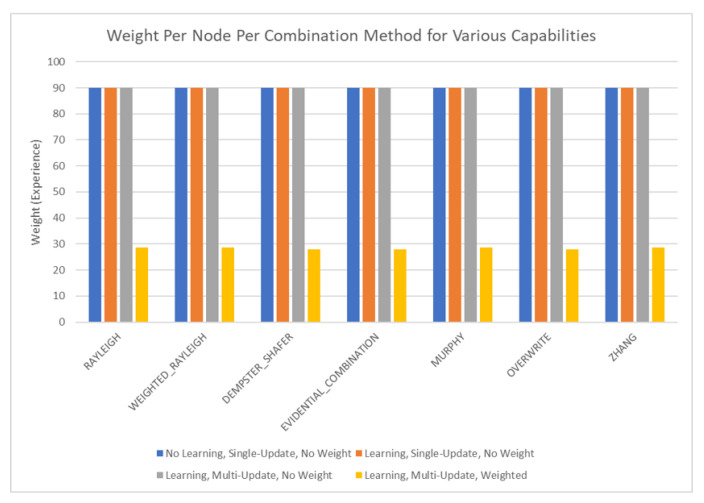
Test results for weighted versus unweighted methods in a single parent network after 30 updates of 1 unit of experience each. This test shows a clear difference between unweighted methods (90 units of experience per node), and the weighted methods (approximately 30 units of experience per node). The unweighted methods clearly suggest that data is reused. While not the case (each propagated evidence set is from a different observation), this result is less explainable than the weighted methods, calling into question the ability for the network results to be accepted in decision-making scenarios.

The results of these tests are shown in Figure 10, Figure 11, Figure 12 and Figure 13. Figure 10, showing the time test results, emphasizes that the weighting methodology helps to overcome the additional burden of the learning calculations. In all combination algorithms, weighting improves the update time to only [1,2] orders of magnitude greater than the no-learning overwrite case, which is the baseline ($10^{-3}$ and $10^{-4}$ versus $10^{-5}$). Compared with the unweighted cases, which are always in the mid to high $10^{-3}$ order of magnitude, this is a significant improvement, thus potentially enabling single-parent network implementation in a real-time embedded system. In the second result, Figure 11, the learning method returns an obvious improvement over all non-learning methods, resulting in effectively perfect consistency with round-off error and a high degree of explainability. In the third result, Figure 12, the three learning cases clearly improve upon the unknown knowledge in the network, even given that the no-learning cases started with a high degree of known transition potentials, and the learning cases started with fully known information. This result is dependent upon the number of evidence sets injected, but the potential to reduce unknown information is shown clearly through this test. The final result, Figure 13, is perhaps the most straight-forward. The weighting method clearly shows approximately 30 units of experience/weight for 30 updates of 1 unit of experience, as expected. All unweighted methods show 3X units of experience per node, which suggests information was used 3 times for each update. This result brings into question the validity of results obtained from networks using unweighted updates since reusing the same information in DS logic changes the results.

### 6.4. Multi-Parent Learning Results

**Figure 14:**
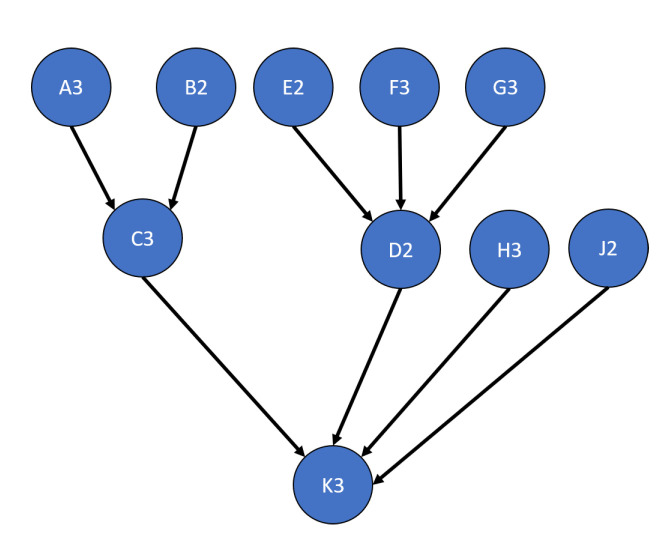
Test network used to analyze the performance of the novel Dempster-Shafer network algorithms. This network only includes multiple parent nodes for each child node. The number after the node name shows the number of θs for the node. Two and three θs nodes were used since these are the more common cases for nodes in a DS network.

**Figure 15:**
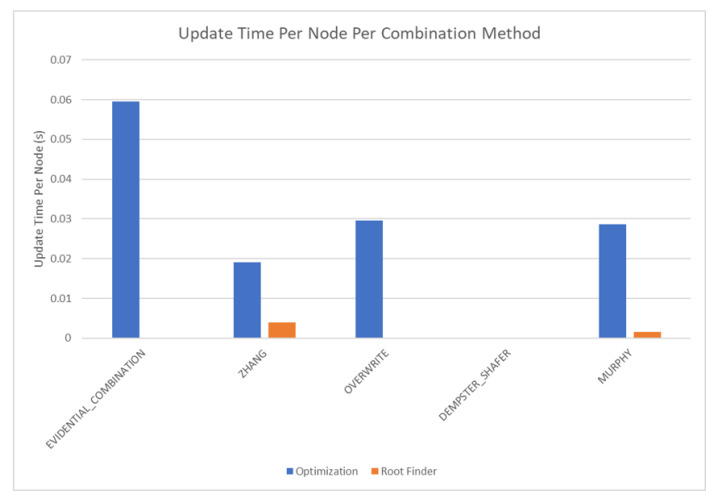
Test results for run time with a multiple parent network. The root finder primarily works for Murphy’s and Zhang’s combination methods, as expected. For those methods, the root finder shows at least an order of magnitude improvement in run time per node, which translates to significant improvements for larger networks.

**Figure 16:**
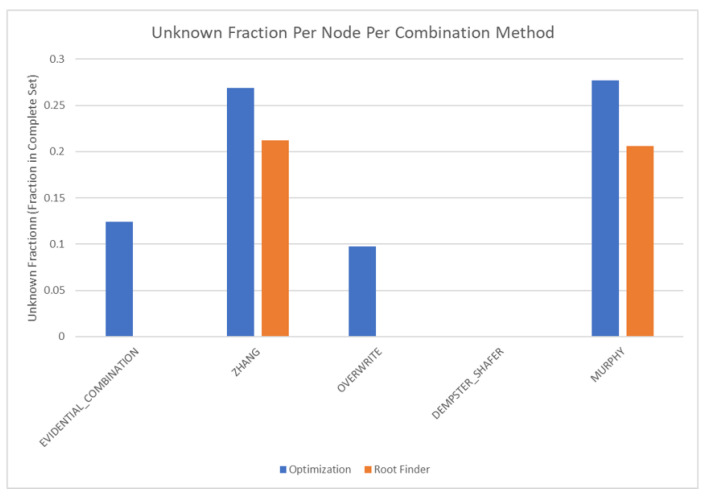
Test results for learning with a multi-parent network. The unknown fraction are similar between the optimization and root finder methods. This result is expected, given that similar solutions should be found. Notably, significantly higher unknown fractions are found for the multiple parent cases than for the single parent cases. This difference is due to the learning method since identical marginals are first calculated for each parent, resulting in duplicated unknown information being retained significantly longer.

**Figure 17:**
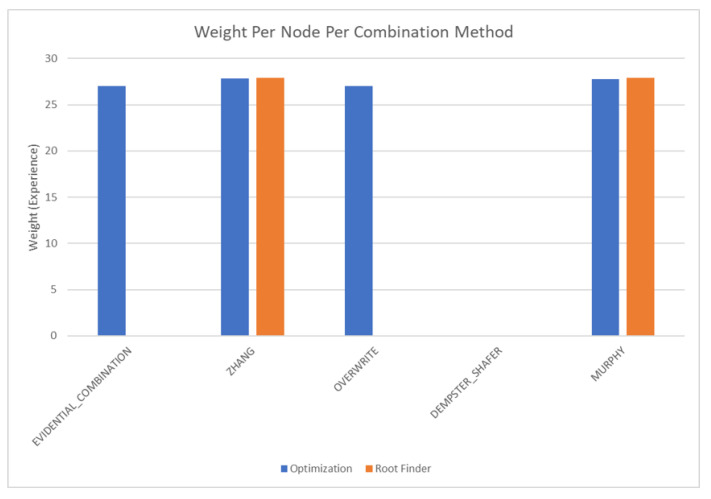
Test results for weighting with multi-parent networks. Since all cases are weighted, no deviations between cases were expected or observed. All cases show expected total weights per node of approximately 30, confirming the results from the single parent test case in Figure 13.

**Figure 18:**
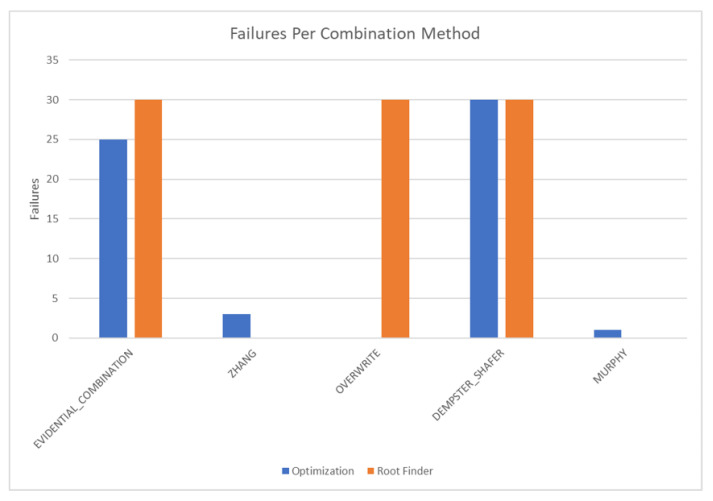
Test failures for the multiple parent cases. The ECR method, Dempster’s Rule, and the overwrite method were not expected to reliably succeed due to the random evidence sets that were not within bounds required for the reverse solver method to succeed. As expected, these methods tend to fail. The root finder method more consistently succeeds or fails. In each set of tests, the root finder method either succeeds or fails in all tests while the optimizer can find solutions that the root finder misses. Murphy’s Rule and Zhang’s Rule show better performance by the root finder than the overwrite method.

Based on the results of the single parent tests, the multi-parent tests are set up to only test learning with weighted simultaneous updates. The additional complexity that these tests add is the need to calculate multiple simultaneous parent marginals through the use of an optimizer or the root finding method detailed in Section 3.3. The test network used is shown in Figure 14. The results are shown in Figure 15, Figure 16, Figure 17 and Figure 18. In most cases, no significant deviation is observed between the optimizer and root finder, especially for the combination methods most likely to be used. The exception to this statement is the run time. The primary reason for developing the root finder is that it is significantly faster than the optimizer (at least one order of magnitude per node), and it is deterministic, especially for two and three option nodes requiring quadratic or cubic solutions, which are closed form. For a real-time implementation on an embedded system, the root finder can be reliably scheduled and interrupted as necessary while operating in a way that is difficult for generic optimizers. The consistency result is not shown in graphical form since the results were all within round-off error of zero, demonstrating perfect consistency, which aligns with the results from the single parent case in Figure 11.

### 6.5. Complex Network Results

**Figure 19:**
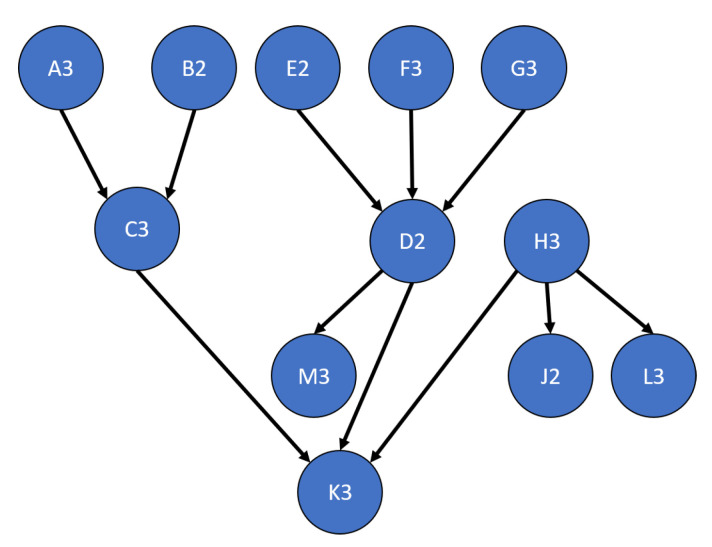
Test network used to analyze the performance of the novel Dempster-Shafer network algorithms. This example includes nodes that have both single and multiple parents. The number after the node name shows the number of θs for the node. Two and three θs nodes were used since these are the more common cases for nodes in a DS network.

**Figure 20:**
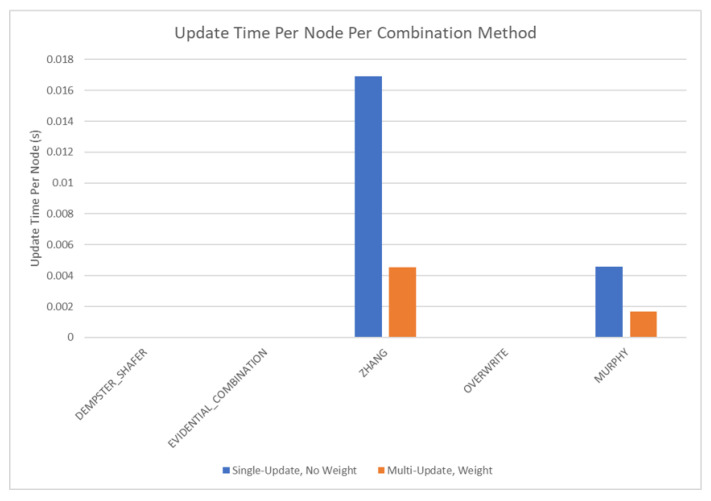
Test results for run time for a complex network. In both cases which succeeded, the weighting method significantly decreased run time, as expected. In both cases, the run time order of magnitude more closely resembles the multiple parent tests (Figure 15) than the single parent tests (Figure 10). This is expected, given that the complex network adds the additional multi-parent calculations. These results also suggest that the root finding method for multi-parents still dominates the single parent solution method.

**Figure 21:**
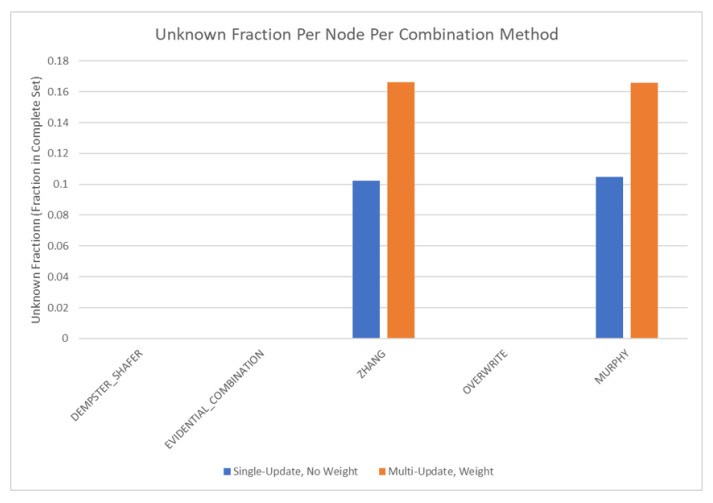
Test results for learning for a complex network. There are two points of interest here: i. The weighted, multiple update method does display a higher unknown fraction. This opposes the results seen in the single-parent tests (Figure 12), suggesting that the multiple parent solution method fares less well when dealing with weighted data; ii. The unknown fraction is between the single parent tests (Figure 12), and the multi parent tests (Figure 16), which is expected, given that the complex network is a combination of the previous networks.

The final tests are performed on the complex network shown in Figure 19, and the results are shown in Figure 20 and Figure 21. Within these tests, the comparison is made between unweighted single updates and weighted multiple simultaneous updates. In this case, it is also instructive to compare these results with the previous tests. Overall, the complex test case falls between the single parent and multi-parent test cases, as expected, given that it is a combination of the two. The complex test case is more similar to the multi-parent test case, suggesting that the multi-parent root finding method properties tend to dominate the metrics in these tests. Failure results are not shown in graphical form since the trends add no new information. There were 30 failures for Dempster’s Rule [1], ECR [15], and the Overwrite Rule. There were zero failures for Zhang’s Method [13] and Murphy’s Method [14]. In all cases, the weighting scheme had no effect on the number of failures. Likewise, the weighting results for complex networks were consistent with Figure 13 and Figure 17, suggesting that the weighting results obtained in this section can be extended to DS networks of arbitrary complexity. Finally, the consistency test results for the complex network are not shown since all tested cases resulted in effectively zero, demonstrating perfect consistency in line with the results from the single and multi-parent solutions (Figure 11).

### 6.6. Episodic Learning Evaluation

Tests for episodic learning were conducted to determine whether this learning method better captured expected relationships than the least squared method without episodes. A two-node network was constructed with a parent node and a child node linked through a transition potential matrix. Both nodes and the transition potentials were initialized to unknown and vacuous, respectively. Two equal weight evidence sets were then entered as defined in Table 7. Murphy’s Rule [14] was used to combine the data since Murphy’s Rule [14] provides a simple result, allowing the effects of the episodic learning to be tested. The baseline test case without episodic learning injected the evidence sets to their respective nodes and update the transition potentials matrix after each update. The results are shown in Table 8. The network was then reset, and the episodic test case was run, with the two evidence sets injected to their respective nodes. In this case, the two evidence sets (“Evidence 1” and “Evidence 2”) were considered to be different episodes, and the episodic learning algorithm was run. The results are shown in Table 8. As can be seen by comparing the values in the table, the episodic learning adjusted the weights in the transition potential matrix to a more intuitive result based on our expectations, given the matching evidence sets.

## 7. Conclusions

The new rules created for the DS network updates significantly changed how the networks function. By requiring a combination algorithm to be used at each node, similar to previous works, simultaneous evidence can be entered, allowing the transition potentials to be calculated from the evidence. This relies on the concept of consistency in the network—that transition potentials both define the influence between nodes and can be used for exact mathematical relationships. As shown in Section 6, propagation of evidence without learning updates to the transition potentials results in a significantly less consistent network. While it can be argued that the transition potentials in this case are merely measures of influence and do not require consistency, the breakdown in the mathematical structure of the network means that important properties are lost, such as the ability to reconstruct the network from partial information. There are some limiting assumptions made, primarily for multi-parent nodes. In these cases, the assumption of identical evidence inputs from the parent nodes along with the limitation to certain DS combination algorithms limits the novel algorithm developments from being used in all scenarios. However, the DS combination algorithms used can be applied to most scenarios through appropriate network definition, as discussed in Section 3.3. The identical evidence assumption is reasonable for many scenarios since this assumption is only made while updating the transition potentials and aligns with the initial conditions of unknown marginals and vacuous transition potential matrices. Further, that restriction only applies when using the polynomial solution; a higher order optimizer can relax that assumption. Future research trajectories include relaxing this assumption without the use of higher order optimizers.

Beyond the consistency obtained through updating the transition potentials, the learning mechanism enables starting from a completely unknown set of data to fill in the entire network. As shown in Section 6, learned networks with random input data results in better known networks than those with *a priori* data provided. The *a priori* data provided is based on assumptions of data that would be available. That *a priori* data could serve as a starting point for learning, resulting in a better known network in all cases. The transition update rules defined in this chapter capture relationships when the entire belief distributions can be considered simultaneously. For scenarios in which this is not the case, episodic learning was introduced to focus the transition updates on the portion of the belief distribution that was observed during the evidence, borrowing from the well-known concept of observability in control theory [30]. This novel use of episodes for updating Dempster-Shafer network transitions significantly improved how the relationships between nodes were captured, as shown in Section 6.6. Future research trajectories include using statistical tests to determine breaks between episodes.

The evidence weighting has a considerable impact on the updates to the network. Speed improved considerably since propagation was not required to all nodes in the network. Furthermore, the automatic handling of reliability of propagated evidence versus observed evidence enables more reliable results. Finally, an accurate representation of the accumulated evidence in the network enables testable results since they can be benchmarked against a consistent basis. While evidence weighting based on inference versus direct observations has already been implemented [3], Section 5 codifies explicit rules for calculating the weights from evidence entry in the network, creating a consistent basis for updates with clearly defined reasoning.

Combining these rules together results in decreased requirements for subject matter expert’s inputs. As detailed in Section 2.3, previous methods have data requirements of all values in every transition matrix, updated each time the matrices change. Using the novel rules defined in this paper, the subject matter expert must define the combination method used at each node, weighting scheme used, episodes (or method used to distinguish episodes), and the control value used in episodic learning. These requirements are different than the original requirements; more importantly, these values can be set once instead of on each update as previously required, resulting in a significant reduction in user input.

As detailed in Section 3, the proposed update rules do not require the specific novel algorithm developed for learning the transition matrices. Use of the novel algorithm enables transition matrix updates with limited computation resources and time. This combination of novel algorithm and update rules enables a wide use of auto-updating DS networks.

Together, these rules realize a capability which was envisioned in the 1980s [7], extended through multiple works in the 1990s and 2000s, and brought to fruition through this work. While valuation networks provide an enormously capable system for understanding and evaluating knowledge of the world, this work enables starting from unknown or limited information and learning relationships without requiring significant subject matter expert’s inputs—a capability which allows reasoning about the world to progress quickly and with reduced guidance from experts.

## Figures and Tables

**Table 1:** Dempster-Shafer Evidence Example. The “Powerset” column represents the full set of options to which a BPA can be assigned. The “Evidence 1” column shows the first evidence set from the sensor that distinguishes shape. The “‘Evidence 2” column shows a second evidence from a sensor that distinguishes color. The “Combined” column shows the rounded, combined masses based on Dempster’s Rule, and the “Bel” and “Pl” columns show the Belief and Plausibility functions, respectively, for each of the elements of the powerset of the combined data.

Powerset	Evidence 1	Evidence 2	Combined	Bel	Pl
Red ball	0.0	0.0	0.490	0.490	0.734
Green ball	0.0	0.2	0.184	0.184	0.367
Red cube	0.1	0.0	0.082	0.082	0.163
(Red ball, Green ball)	0.8	0.0	0.163	0.837	0.857
(Green ball, Red cube)	0.0	0.0	0.000	0.266	0.286
(Red ball, Red cube)	0.0	0.6	0.061	0.633	0.653
(Red ball, Green ball, Red cube)	0.1	0.2	0.020	1.0	1.0

**Table 2:** Bayesian Probability Example. This example mirrors the Dempster-Shafer (DS) example in Table 1 as closely as possible for comparison. Because the evidences are direct observations of the priors, the likelihood is 1.0.

Θ	Prior	Likelihood 1	Posterior	Likelihood 2	Posterior
Red ball	0.333		0.459		0.623
Green ball	0.333		0.459		0.267
Red cube	0.333		0.081		0.110
Incorrect shape		0.15			
Correct shape		0.85			
Incorrect color				0.3	
Correct color				0.7	

**Table 3:** Dempster-Shafer Conflicting Example. The combination of highly conflicting data provides non-intuitive results. In this case, although both A and C each have a large belief mass in an evidence set, 0 mass for each of A and C in the other evidence set results in a vote-no-by-one scenario in which one sensor “votes no” for A and the other sensor “votes no” for C. The result is that all belief mass is given to B when combined. Note that for this simple example, only single options are focal points in the frame of discernment.

Data Set	A	B	C	(A,B)	(A,C)	(B,C)	(A,B,C)
Evidence 1	0.9	0.1	0.0	0.0	0.0	0.0	0.0
Evidence 2	0.0	0.1	0.9	0.0	0.0	0.0	0.0
Combination	0.0	1.0	0.0	0.0	0.0	0.0	0.0

**Table 4:** Dempster-Shafer combination details for two identical input evidence sets with three options each. E1 and E2 are evidence sets one and two, respectively. Each matrix cell mass is assigned to the specified Destination row mass unless otherwise stated in the cell.

					E1			
		*A*	*B*	*C*	$\left(A,B\right)$	$\left(A,C\right)$	$\left(B,C\right)$	$\left(A,B,C\right)$
	*A*	$A^{2}$	0	0	$\left(A,B\right)A\to A$	$\left(A,C\right)A\to A$	0	$\left(A,B,C\right)A\to A$
	*B*	0	$B^{2}$	0	$\left(A,B\right)B\to B$	0	$\left(B,C\right)B\to B$	$\left(A,B,C\right)B\to B$
E2	*C*	0	0	$C^{2}$	0	$\left(A,C\right)C\to C$	$\left(B,C\right)C\to C$	$\left(A,B,C\right)C\to C$
	$\left(A,B\right)$	$\left(A,B\right)A$	$\left(A,B\right)B$	0	${\left(A,B\right)}^{2}$	$\left(A,B\right)\left(A,C\right)\to A$	$\left(A,B\right)\left(B,C\right)\to B$	$\left(A,B,C\right)\left(A,B\right)\to \left(A,B\right)$
	$\left(A,C\right)$	$\left(A,C\right)A$	0	$\left(A,C\right)C$	$\left(A,C\right)\left(A,B\right)\to A$	${\left(A,C\right)}^{2}$	$\left(A,C\right)\left(B,C\right)\to C$	$\left(A,B,C\right)\left(A,C\right)\to \left(A,C\right)$
	$\left(B,C\right)$	0	$\left(B,C\right)B$	$\left(B,C\right)C$	$\left(B,C\right)\left(A,B\right)\to B$	$\left(B,C\right)\left(A,C\right)\to C$	${\left(B,C\right)}^{2}$	$\left(A,B,C\right)\left(B,C\right)\to \left(B,C\right)$
	$\left(A,B,C\right)$	$\left(A,B,C\right)A$	$\left(A,B,C\right)B$	$\left(A,B,C\right)C$	$\left(A,B,C\right)\left(A,B\right)$	$\left(A,B,C\right)\left(A,C\right)$	$\left(A,B,C\right)\left(B,C\right)$	${\left(A,B,C\right)}^{2}$
	Destination	→A	→B	→C	$\to \left(A,B\right)$	$\to \left(A,C\right)$	$\to \left(B,C\right)$	$\to \left(A,B,C\right)$

**Table 5:** Expected relationships between the cross-traffic light and the time until green. Given knowledge of the cross-traffic light at an intersection, this table details the expectations of the time until the light changes from red to green for the evaluator to continue through the intersection. Note that any ambiguous sets are removed for ease of description. Those sets can be interpolated from the relationships shown in this table.

	Green	Yellow	Red
Long	0.9	0.1	0.0
Medium	0.1	0.8	0.1
Short	0.0	0.1	0.9

**Table 6:** Example of why additional weighting schemes are necessary. “Evidence 1” has significantly higher weight than “Evidence 2”. Without an included weighing scheme, the resulting combined data in “Rayleigh” and “Murphy” have a balance in potential solutions between “A” and “C” instead of heavily favoring “A”, which would be expected if only “Evidence 1” were combined through the DS combination methods. The resulting combined data in “Weighted Rayleigh” and “Weighted Murphy” favor “A”, which is expected since “Evidence 1” favors “A”. Note that the Rayleigh [28] method is designed to amplify decisions; thus a combination comprised of “Evidence 1” multiple times would be expected to nearly exclusively return “A”.

Data Set	Experience	A	B	C	(A,B)	(A,C)	(B,C)	(A,B,C)
Evidence 1	30.0	0.5	0.1	0.2	0.0	0.05	0.05	0.1
Evidence 2	0.5	0.1	0.05	0.4	0.05	0.2	0.1	0.1
Rayleigh	n/a	0.47	6.1 × 10${}^{-4}$	0.24	2.6 × 10${}^{-4}$	0.28	1.2 × 10${}^{-3}$	2.2 × 10${}^{-4}$
Weighted Rayleigh	30.5	0.91	3.8 × 10${}^{-5}$	2.2 × 10${}^{-2}$	1.4 × 10${}^{-5}$	6.9 × 10${}^{-2}$	1.6 × 10${}^{-4}$	2.2 × 10${}^{-3}$
Murphy	n/a	0.30	0.03	0.30	0.025	0.13	0.075	0.10
Weighted Murphy	30.5	0.49	0.011	0.20	8.2 × 10${}^{-4}$	5.2 × 10${}^{-2}$	5.1 × 10${}^{-2}$	0.10

**Table 7:** Episodic Learning Test. The following two evidence sets have distinctly different correlations for the two-node network. “Evidence 1” shows a correlation between “Option A” in the parent node and “Option C” in the child node, and “Evidence 2” shows a correlation between “Option B” in the parent node and “Option D” in the child node.

Data Set	Option_A	Option_B	(Option_A, Option_B)	Option_C	Option_D	(Option_C, Option_D)
Evidence 1	0.9	0.08	0.02	0.9	0.1	0.0
Evidence 2	0.05	0.9	0.05	0.1	0.7	0.2

**Table 8:** Episodic Learning Results. The baseline without episodic learning started with unknown information (all marginal masses in the complete sets). Both evidence sets were added and combined via Murphy’s Rule [14] into their respective nodes, and the transition potential matrix was updated after each evidence injection. The second test applied the “Evidence 1” sets to the appropriate nodes and subsequently ran the transition potential matrix update algorithm. The node marginals were then reset to the unknown state, and the “Evidence 2” sets were applied to the appropriate nodes. The transition potential matrix update algorithm was again run to incorporate the second episode.

**Without Episodic**	**Option_A**	**Option_B**	**(Option_A, Option_B)**
Option_C	0.552	0.455	0.821
Option_D	0.448	0.329	0.179
(Option_C, Option_D)	0.0	0.216	0.0
**With Episodic**	**Option_A**	**Option_B**	**(Option_A, Option_B)**
Option_C	0.727	0.0	0.233
Option_D	0.129	0.703	0.336
(Option_C, Option_D)	0.143	0.297	0.431

## Nomenclature

Belief Mass	A non-negative measure similar to probabilities, but a non-classical idea in which the masses are not necessarily based on the occurrence of an event.
Frame of Discernment $\left(\Theta \right)$	A set of mutually exclusive elements. Belief masses can be assigned to these elements or to sets of these elements.
Powerset ($2^{\Theta }$)	The set of all subsets of Θ. If there are *n* elements in Θ, then there are $2^{n}$ elements in the powerset of Θ.
Basic Probability Assignment $\left(BPA\right)$	An assignment of a belief mass in the range $\left[0,1\right]$ to each element of the powerset. $m:2^{\Theta }\to \left[0,1\right]$, where $m\left(\emptyset \right)=0$, $\sum m\left(A\right)\geq 0$, A∈powerset. If $\sum m\left(A\right)=1$ and the masses are based on the occurrence of an event, then these masses are equivalent to probabilities. Subsequently, we will assume $\sum m\left(A\right)=1$.
Evidence	An observation described by a BPA.
Focal Point	Any subset of the powerset to which a belief mass of greater than zero is assigned. $A\in powerset|m\left(A\right)>0$
Belief Function $\left(Bel\right)$	Given a BPA with $\left\{A_{1},\ldots ,A_{n}\right\}\in 2^{\Theta }$ and $A_{x}\in \Theta$, $Bel\left(A_{x}\right)=\sum_{A_{x}\subseteq A}m\left(A_{i}\right)$.
Plausibility Function $\left(Pl\right)$	Given a BPA with $\left\{m\left(A_{1}\right),\ldots ,m\left(A_{n}\right)\right\}\in 2^{\Theta }$ and $A_{x}\in \Theta$, $Pl\left(A_{x}\right)=1-\sum_{A\cap A_{x}=\emptyset }m\left(A_{i}\right)$.
Dempster’s Rule	The original rule proposed by Arthur Dempster [1] for combining evidence: $\left(m_{1}⊕m_{2}\right)\left(x\right)=\frac{\sum_{E\cap E^{{}^{\prime }}=x}m_{1}\left(E\right)m_{2}\left(E^{{}^{\prime }}\right)}{1-\sum_{E\cap E^{{}^{\prime }}=\emptyset }m_{1}\left(E\right)m_{2}\left(E^{{}^{\prime }}\right)}$.

## Appendix A. Dempster-Shafer Multi-Parent Reverse Solver Restriction Validation

In this appendix, we develop the result necessary to enable restricting the inputs to Dempster’s Rule and ECR that enable calculating a valid solution to the reverse solver for multiple parents. The following assumption still applies: for a valid solution to be guaranteed, there must be at least as many evidence inputs as parent marginals for which the algorithm is solving, not including evidence inputs that contain all mass in the universal set.

Furthermore, the following simplifying assumptions enable easier analysis while still applying to the general result:

- ECR is only valid for this result when all weights and reliabilities are equal, thus reducing ECR to Dempster’s Rule.
- The result is only required to be developed for two evidence inputs. This assumption is because both Dempster’s Rule and ECR add the new evidence into the combined result of the old evidence. Thus, if the combined result provides a valid reverse solution method, then combining any new evidence using the same restrictions will in itself be a combination of two evidences under the same restrictions, resulting in a recursive proof.
- This result is only shown for a three option evidence set (a, b, c) and the associated power set. The reason for this can be seen through the solution method developed in Section 3.3. Each solved mass only depends on the masses of higher ambiguity which apply to the solution mass. Thus, the shown proof is, again, recursive.

Recall from Section 3.3, the child marginal *a* in terms of two identical sets of parent marginals is given by Equation (A1).

(A1) $$a_{c}=a_{p}^{2}+2*a_{p}\left[{\left(a,b\right)}_{p}+{\left(a,c\right)}_{p}+{\left(a,b,c\right)}_{p}\right]+2*{\left(a,b\right)}_{p}{\left(a,c\right)}_{p}$$

To solve for $a_{p}$, the quadratic formula is used to give Equation (A2).

(A2) $$\begin{array}{c} a_{p}=-\left[{\left(a,b\right)}_{p}+{\left(a,c\right)}_{p}+{\left(a,b,c\right)}_{p}\right]+\\ \sqrt{{\left[{\left(a,b\right)}_{p}+{\left(a,c\right)}_{p}+{\left(a,b,c\right)}_{p}\right]}^{2}-\left(2*{\left(a,b\right)}_{p}{\left(a,c\right)}_{p}-a_{c}\right)} \end{array}$$

From Equation (A2), it is quite clear that a valid, positive solution will be obtained provided that Equation (A3) holds.

(A3) $$a_{c}\geq 2*{\left(a,b\right)}_{p}{\left(a,c\right)}_{p}$$

However, this solution specifically applies to the cases in which the two parent evidence sets are not identical. Thus, from Table 4, $a_{c}$ can be calculated through Equation (A4) where ${\left(\;\right)}_{1}$ denotes the first evidence set and ${\left(\;\right)}_{2}$ denotes the second evidence set.

(A4) $$\begin{array}{c} a_{c}=a_{1}*a_{2}+{\left(\left(a,b\right)+\left(a,c\right)+\left(a,b,c\right)\right)}_{1}*a_{2}+\\ {\left(\left(a,b\right)+\left(a,c\right)+\left(a,b,c\right)\right)}_{2}*a_{1}+{\left(a,b\right)}_{1}*{\left(a,c\right)}_{2}+{\left(a,b\right)}_{2}*{\left(a,c\right)}_{1} \end{array}$$

Likewise for the left side of the inequality, the following relationships hold:

(A5) $${\left(a,b,c\right)}_{p}=\sqrt{{\left(a,b,c\right)}_{c}}$$

(A6) $${\left(a,b\right)}_{p}=-{\left(a,b,c\right)}_{p}+\sqrt{{\left(a,b,c\right)}_{p}^{2}+{\left(a,b\right)}_{c}}$$

Recalling further from Table 4 the following two relationships:

(A7) $${\left(a,b\right)}_{c}={\left(a,b,c\right)}_{1}{\left(a,b\right)}_{2}+{\left(a,b,c\right)}_{2}{\left(a,b\right)}_{1}+{\left(a,b\right)}_{1}{\left(a,b\right)}_{2}$$

(A8) $${\left(a,b,c\right)}_{c}={\left(a,b,c\right)}_{1}{\left(a,b,c\right)}_{2}$$

With substitution, Equations (A4)–(A8) result in Equation (A9).

(A9) $$\begin{array}{c} 2\left(-\sqrt{{\left(a,b,c\right)}_{1}{\left(a,b,c\right)}_{2}}\right.\\ \left.+\sqrt{{\left(a,b,c\right)}_{1}{\left(a,b,c\right)}_{2}+{\left(a,b,c\right)}_{1}{\left(a,b\right)}_{2}+{\left(a,b,c\right)}_{2}{\left(a,b\right)}_{1}+{\left(a,b\right)}_{1}{\left(a,b\right)}_{2}}\right)\\ \left(-\sqrt{{\left(a,b,c\right)}_{1}{\left(a,b,c\right)}_{2}}\right.\\ \left.+\sqrt{{\left(a,b,c\right)}_{1}{\left(a,b,c\right)}_{2}+{\left(a,b,c\right)}_{1}{\left(a,c\right)}_{2}+{\left(a,b,c\right)}_{2}{\left(a,c\right)}_{1}+{\left(a,c\right)}_{1}{\left(a,c\right)}_{2}}\right)\leq \\ a_{1}*a_{2}+{\left(\left(a,b\right)+\left(a,c\right)+\left(a,b,c\right)\right)}_{1}*a_{2}+\\ {\left(\left(a,b\right)+\left(a,c\right)+\left(a,b,c\right)\right)}_{2}*a_{1}+{\left(a,b\right)}_{1}*{\left(a,c\right)}_{2}+{\left(a,b\right)}_{2}*{\left(a,c\right)}_{1} \end{array}$$

Next, we simplify the left side of Equation (A9) slightly. Note that $\sqrt{a+b}\leq \sqrt{a}+\sqrt{b}$. Therefore, Equation (A10) holds, and Equation (A11) can be used to constrain the left side of Equation (A9).

(A10) $$\begin{array}{c} \sqrt{{\left(a,b,c\right)}_{1}{\left(a,b,c\right)}_{2}+{\left(a,b,c\right)}_{1}{\left(a,b\right)}_{2}+{\left(a,b,c\right)}_{2}{\left(a,b\right)}_{1}+{\left(a,b\right)}_{1}{\left(a,b\right)}_{2}}\leq \\ \sqrt{{\left(a,b,c\right)}_{1}{\left(a,b,c\right)}_{2}}+\\ \sqrt{{\left(a,b,c\right)}_{1}{\left(a,b\right)}_{2}+{\left(a,b,c\right)}_{2}{\left(a,b\right)}_{1}+{\left(a,b\right)}_{1}{\left(a,b\right)}_{2}} \end{array}$$

(A11) $$\begin{array}{c} 2\left(-\sqrt{{\left(a,b,c\right)}_{1}{\left(a,b,c\right)}_{2}}\right.\\ \left.+\sqrt{{\left(a,b,c\right)}_{1}{\left(a,b,c\right)}_{2}+{\left(a,b,c\right)}_{1}{\left(a,b\right)}_{2}+{\left(a,b,c\right)}_{2}{\left(a,b\right)}_{1}+{\left(a,b\right)}_{1}{\left(a,b\right)}_{2}}\right)\\ \left(-\sqrt{{\left(a,b,c\right)}_{1}{\left(a,b,c\right)}_{2}}\right.\\ \left.+\sqrt{{\left(a,b,c\right)}_{1}{\left(a,b,c\right)}_{2}+{\left(a,b,c\right)}_{1}{\left(a,c\right)}_{2}+{\left(a,b,c\right)}_{2}{\left(a,c\right)}_{1}+{\left(a,c\right)}_{1}{\left(a,c\right)}_{2}}\right)\leq \\ 2\left(\sqrt{{\left(a,b,c\right)}_{1}{\left(a,b\right)}_{2}+{\left(a,b,c\right)}_{2}{\left(a,b\right)}_{1}+{\left(a,b\right)}_{1}{\left(a,b\right)}_{2}}\right)\\ \left(\sqrt{{\left(a,b,c\right)}_{1}{\left(a,c\right)}_{2}+{\left(a,b,c\right)}_{2}{\left(a,c\right)}_{1}+{\left(a,c\right)}_{1}{\left(a,c\right)}_{2}}\right) \end{array}$$

Thus, if we show that the right side of Equation (A11) is less than or equal to the left side of Equation (A9), then Equation (A9) holds, and the restrictions will satisfy the constraint necessary to ensure a solution to the reverse solver. To do this, we introduce the restrictions from Equations (9) and (10). Further, without loss of generality, we assume that ${\left(a,b\right)}_{1}\geq {\left(a,b\right)}_{2}$, ${\left(a,c\right)}_{1}\geq {\left(a,c\right)}_{2}$, ${\left(a,b\right)}_{1}\geq {\left(a,c\right)}_{1}$, and ${\left(a,b\right)}_{2}\geq {\left(a,c\right)}_{2}$.

Using Equation (9) for $a_{1}$ and $a_{2}$ the least constraining choices of ${\left(a,b\right)}_{1}$, ${\left(a,b\right)}_{2}$, ${\left(a,c\right)}_{1}$, and ${\left(a,c\right)}_{2}$, the right side of Equation (A9) can be constrained via Equation (A12), which also includes a square and square root to end up with the same form as the right side of Equation (A11).

(A12) $$\begin{array}{c} \left(\left(3{\left(a,b\right)}_{1}{\left(a,b\right)}_{2}+{\left(a,b,c\right)}_{1}{\left(a,b\right)}_{2}+{\left(a,b,c\right)}_{2}{\left(a,b\right)}_{1}\right.\right.\\ {\left.{\left.+2{\left(a,c\right)}_{1}{\left(a,b\right)}_{2}+2{\left(a,c\right)}_{2}{\left(a,b\right)}_{1}\right)}^{2}\right)}^{\frac{1}{2}}\leq \\ a_{1}*a_{2}+{\left(\left(a,b\right)+\left(a,c\right)+\left(a,b,c\right)\right)}_{1}*a_{2}+\\ {\left(\left(a,b\right)+\left(a,c\right)+\left(a,b,c\right)\right)}_{2}*a_{1}+{\left(a,b\right)}_{1}*{\left(a,c\right)}_{2}+{\left(a,b\right)}_{2}*{\left(a,c\right)}_{1} \end{array}$$

Using the restrictions from Equation (10), we cancel terms and end up with Equation (A13).

(A13) $$\begin{array}{c} 2\left(\sqrt{{\left(a,b,c\right)}_{1}{\left(a,b\right)}_{2}+{\left(a,b,c\right)}_{2}{\left(a,b\right)}_{1}+{\left(a,b\right)}_{1}{\left(a,b\right)}_{2}}\right)\\ \left(\sqrt{{\left(a,b,c\right)}_{1}{\left(a,c\right)}_{2}+{\left(a,b,c\right)}_{2}{\left(a,c\right)}_{1}+{\left(a,c\right)}_{1}{\left(a,c\right)}_{2}}\right)\leq \\ \left(\left(3{\left(a,b\right)}_{1}{\left(a,b\right)}_{2}+{\left(a,b,c\right)}_{1}{\left(a,b\right)}_{2}+{\left(a,b,c\right)}_{2}{\left(a,b\right)}_{1}\right.\right.\\ {\left.{\left.+2{\left(a,c\right)}_{1}{\left(a,b\right)}_{2}+2{\left(a,c\right)}_{2}{\left(a,b\right)}_{1}\right)}^{2}\right)}^{\frac{1}{2}} \end{array}$$

Therefore, the original inequality in Equation (A3) is true, and the restricted set is shown to have a valid reverse solution via the method developed in Section 3.3.

## Appendix B. Dempster-Shafer Algorithm Weighting Modifications

Other than the ECR method [15], Dempster’s Rule and rules created to replace/improve Dempster’s Rule consider all evidence equally, without weight. Due to the modifications in Figure 8, it was necessary to introduce weights to algorithms which could be used in the Dempster-Shafer network. Murphy’s Rule [14] and the two Rayleigh methods [28] were easily modified to include weight by weighing the evidential inputs. For Murphy’s Rule, this equates to a weighted average. For the Rayleigh methods, this means that evidential masses are multiplied by the associated weights when combined with the other evidential masses, whether they are supportive or in conflict. Zhang’s Rule [13] had to be handled differently since multiplying the input evidence masses by the weights do not effect the result because the first use of the evidence masses is to calculate conflict. The conflict calculation uses a dot product, which is normalized, thus removing the weighting. Instead, the weighted average credibility of the original reliability was modified to use both the weighting developed through Zhang’s support calculations and the input evidence weights, thus affecting the final result through the evidence input weights.

## Appendix C. Dempster-Shafer Transition Solution Alternate Methods

The solution to solve the transitions in a Dempster-Shafer network, as detailed in Section 3.2, was chosen to be least squares with modifications to the solution when the non-negative constraint on all transition values was violated. Alternate solution methods are available that could satisfy the design requirements of low computation requirements and fast solutions. In particular, three options were discussed and are recommended for future evaluation, and a fourth option was evaluated but determined to be unreliable in practice.

- Moore-Penrose Pseudo-Inverse [31]. The definition of the transition in the Dempster-Shafer hypertree, as given in [7], provides the child-to-parent transition values using the transpose of the parent-to-child transition values. This is potentially not the only available definition. An inverse is impossible, given that many cases result in a non-invertible matrix. Figure 4 is a simple example of this case. However, the generalized inverse, using the Moore-Penrose definition, is not restricted in the same manner. This method was not chosen for current development in order to use the hypertree definition given in [7]. It is recommended in future analysis to compare the Moore-Penrose pseudo-inverse definition with the transpose definition in the Dempster-Shafer hypertree and evaluate which method performs better and provides more intuitive and explainable results.
- Iteration using the separation principle [30]. The separation principle is a well-known concept in control theory that allows the system observer to be designed separately from the system controller because the observer dynamics are sufficiently faster than the controller dynamics that the two parts of the system are effectively decoupled. Similarly, the Dempster-Shafer network is typically updated at a slow rate relative to the update rate of a real-time system. For example, the network is often updated on the order of 1Hz to 10Hz, while real-time controllers usually update significantly faster. Given the relatively slow update rate, it should be possible to design a dynamic transition solution method which has significantly faster dynamics than the network update. Again, it is recommended in future analysis to compare this solution method with the least squares solution method developed in this paper.
- Constrained quadratic optimization. This method is similar to least squares in that it uses a quadratic cost function, but this method also includes constraints such as requiring non-negative values in the design vector. Since this is a fairly well-studied area of optimization, an implementation of this method could be fast enough to perform in real-time. However, since there are no guarantees on maximum iterations to complete, this method was not selected for the current development.
- Linear programming. Linear programming, which automatically guarantees positive semi-definite design variables and returns solutions quickly, was tested as part of the development for this paper. Two modifications were made for linear programming. First, the design variables were changed to be the new transition potentials. Second, the cost function was changed to be the sum of the design variables with weights. Higher weights were placed on design variables that were previously close to zero to approximate the effect of least squares minimization. While this optimization resulted in a different solution than the least squares method, it provided feasible solutions with fast computations, which is necessary for real-time updates. However, in practice, the NumPy [32] implementation of this method was found to be unreliable at finding feasible solutions.
